# Innate immune activation and mitochondrial ROS induce acute and persistent cardiac conduction system dysfunction after COVID-19

**DOI:** 10.1172/jci.insight.193164

**Published:** 2025-12-22

**Authors:** Deepthi Ashok, Ting Liu, Misato Nakanishi-Koakutsu, Joseph Criscione, Meghana Prakash, Alexis Tensfeldt, Byunggik Kim, Bryan Ho, Julian Chow, Morgan Craney, Mark J. Ranek, Brian L. Lin, Kyriakos Papanicolaou, Agnieszka Sidor, D. Brian Foster, Hee Cheol Cho, Andrew Pekosz, Jason Villano, Deok-Ho Kim, Brian O’Rourke

**Affiliations:** 1 Division of Cardiology, Department of Medicine, and; 2 Department of Surgery, Johns Hopkins University School of Medicine, Baltimore, Maryland, USA.; 3 Blalock-Taussig-Thomas Pediatric and Congenital Heart Center, Johns Hopkins Children’s Center, Baltimore, Maryland, USA.; 4 Department of Biomedical Engineering, Johns Hopkins University School of Medicine, Baltimore, Maryland, USA.; 5 Department of Mechanical Engineering and; 6 Department of Chemical and Biomolecular Engineering, Whiting School of Engineering, Johns Hopkins University, Baltimore, Maryland, USA.; 7 Research Animal Resources, Johns Hopkins University School of Medicine, Baltimore, Maryland, USA.; 8 Department of Molecular Microbiology and Immunology, Johns Hopkins Bloomberg School of Public Health, Baltimore, Maryland, USA.; 9 Department of Molecular and Comparative Pathobiology, Johns Hopkins University School of Medicine, Baltimore, Maryland, USA.; 10 Johns Hopkins Center for Microphysiological Systems, Baltimore, Maryland, USA.

**Keywords:** Cardiology, Immunology, Infectious disease, Arrhythmias, Innate immunity, Mitochondria

## Abstract

Cardiac arrhythmias increase during acute SARS-CoV-2 infection and in long COVID syndrome, by unknown mechanisms. This study explored the acute and long-term effects of COVID-19 on cardiac electrophysiology and the cardiac conduction system (CCS) in a hamster model. Electrocardiograms and subpleural pressures were recorded by telemetry for 4 weeks after SARS-CoV-2 infection, and interferon-stimulated gene expression and macrophage infiltration of the CCS were assessed at 4 days and 4 weeks postinfection. COVID-19 induced pronounced tachypnea and cardiac arrhythmias, including bradycardia and persistent atrioventricular block, though no viral protein expression was detected in the heart. Arrhythmias developed rapidly, partially reversed, and then redeveloped, indicating persistent CCS injury. COVID-19 induced cardiac cytokine expression, connexin mislocalization, and CCS macrophage remodeling. Interestingly, sterile innate immune activation by direct cardiac injection of polyinosinic:polycytidylic acid (PIC) induced arrhythmias similar to those of COVID-19. PIC strongly induced cytokine secretion and interferon signaling in hearts, human induced pluripotent stem cell–derived cardiomyocytes, and engineered heart tissues, accompanied by alterations in excitation-contraction coupling. Importantly, the pulmonary and cardiac effects of COVID-19 were blunted by JAK/STAT inhibition or a mitochondrially targeted antioxidant, indicating that SARS-CoV-2 infection indirectly leads to arrhythmias by innate immune activation and redox stress, which could have implications for long COVID syndrome.

## Abstract 2

Long-lasting cardiac arrhythmias linked to innate immune activation and oxidative stress in the absence of direct cardiac viral infection in an animal model of COVID-19.

## Graphical abstract

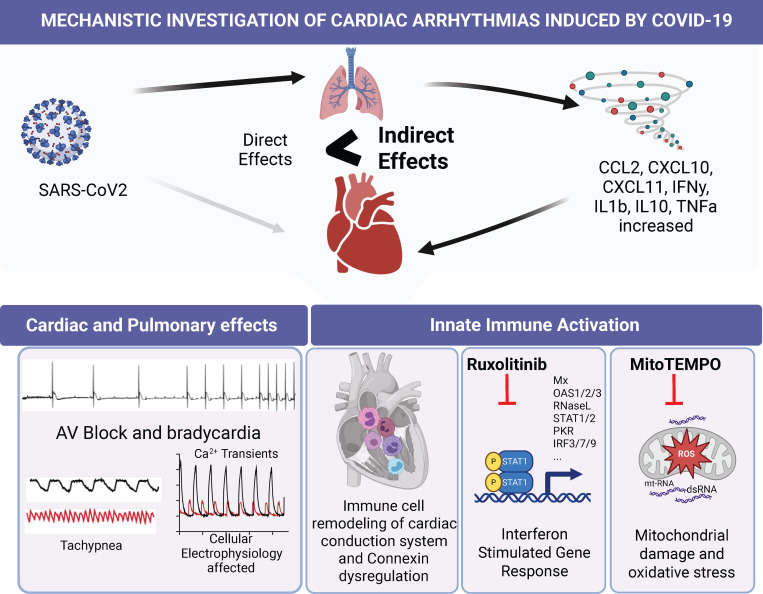

## Introduction

With over 1 million confirmed deaths in the United States and almost 7 million globally, the COVID-19 pandemic uncovered a significant gap in our understanding of virus-host interactions and how to prevent the systemic complications of infection. The specific mechanisms underlying the short- and long-term cardiac consequences of RNA viruses like SARS-CoV-2 are not well understood. Prior to widespread vaccination or natural immunization, adverse cardiac electrophysiological effects were found to be a prominent feature of SARS-CoV-2 infection (1, 2). These included changes in QT interval (3–5); atrial arrhythmias (1, 6–8); bradyarrhythmias, including severe sinus bradycardia and complete heart block (9); ventricular tachycardias (VT); and cardiac arrest (10, 11). In rare cases, cardiac inflammation could also develop or persist after recovery from COVID-19, as reported for collegiate athletes postinfection, even among asymptomatic people (12). When assessed 6 months after SARS-CoV-2 infection, a study of 73,000 patients in the VA hospital system showed a several-fold increased risk of new-onset cardiometabolic disease and arrhythmias compared with uninfected individuals (13). Thus, cardiovascular complications may be an important contributor to SARS-CoV-2 morbidity, even extending to time points encompassing the definition of long COVID syndrome, which is an emergent health problem currently estimated to affect more than 18 million people in the United States (14, 15).

The mechanisms underlying these COVID-19–associated arrhythmias are unknown and could be due to either direct or indirect effects of the virus. For example, during the SARS-CoV-1 outbreak in Toronto in 2002, up to 35% of human heart autopsy samples were positive for SARS-CoV-1 RNA and showed evidence of macrophage infiltration and myocyte necrosis (16). Similarly, several early studies reported SARS-CoV-2 RNA present in postmortem myocardial tissue from patients with COVID-19 (17, 18), though it is unclear whether this represents viral infection or RNA present in interstitial infiltrates from the blood, secondary to vascular leakage (19). A significant fraction (up to 60%) of SARS-CoV-2–infected patients showed evidence of cardiac inflammation or injury by MRI (20), and several studies found evidence of increased plasma IL-6, NT-ProBNP, or troponin T (21–23). Nevertheless, the overall incidence of fulminant myocarditis during or after COVID-19 infection is likely to be low (24), and more recent studies do not support widespread viral replication in the heart (25–28). Even in the absence of direct infection, cardiac interferon-stimulated (IFN-stimulated) gene transcripts significantly increase, indicating that antiviral innate immunity is induced, along with marked changes in mitochondrially encoded genes (26). These data support the idea that SARS-CoV-2 infection can affect the heart by triggering an organism-wide antiviral immune response, but whether its impact is protective or detrimental (as in the “cytokine storm” model) is difficult to ascertain. Outcomes may depend on timing, as early activation of innate immunity inhibits viral replication, but sustained activation might impair function (29).

Reactive oxygen species (ROS) are important mediators of inflammatory and immune cell responses, and increased oxidative stress has been documented for several respiratory viral infections, including COVID-19 (30). Human studies associated serum antioxidant depletion with COVID-19 severity (31, 32), suggesting that antioxidant interventions might prevent or reverse pathogenesis. Several clinical trials were implemented to assess the benefits of antioxidant therapy, including nonenzymatic dietary antioxidants (vitamins A, E, C, zinc, and selenium) (33), reduced glutathione or its precursor N-Acetylcysteine (34–36), nitric oxide (NCT04388683), natural products (37), or synthetic antioxidants (Tempol; NCT04729595). These antioxidant interventions typically decreased cytokines or biomarkers of COVID-19 but had little effect on clinical outcomes. None of the studies were designed to assess effects of the antioxidant treatments on pulmonary or cardiac function in detail or their long-term impact.

Here, we examine the acute and persistent cardiac electrophysiological effects after intranasal SARS-CoV-2 infection in hamsters, while assessing pulmonary function in parallel during the course of COVID-19. Even in the absence of detectable viral protein expression in the heart, we find pronounced expression of IFN-stimulated genes, altered connexin localization, cardiac arrhythmias, and immune cell remodeling in the cardiac conduction system (CCS) and ventricular myocardium. Interestingly, similar arrhythmias could be induced by a mimetic of the viral double-stranded RNA–triggered (dsRNA-triggered) innate immune response in naive animals. Guided by in vitro studies of innate immune activation in human induced pluripotent stem cell–derived cardiomyocyte (hiPSC-CM) monolayers, we further show that in vivo interventions designed to inhibit IFN signaling or mitochondrial ROS mitigate the cardiac and pulmonary effects of COVID-19 in the hamster model.

## Results

### Effects of SARS-CoV-2 infection on systemic and pulmonary function in hamsters.

**Figure 1:**
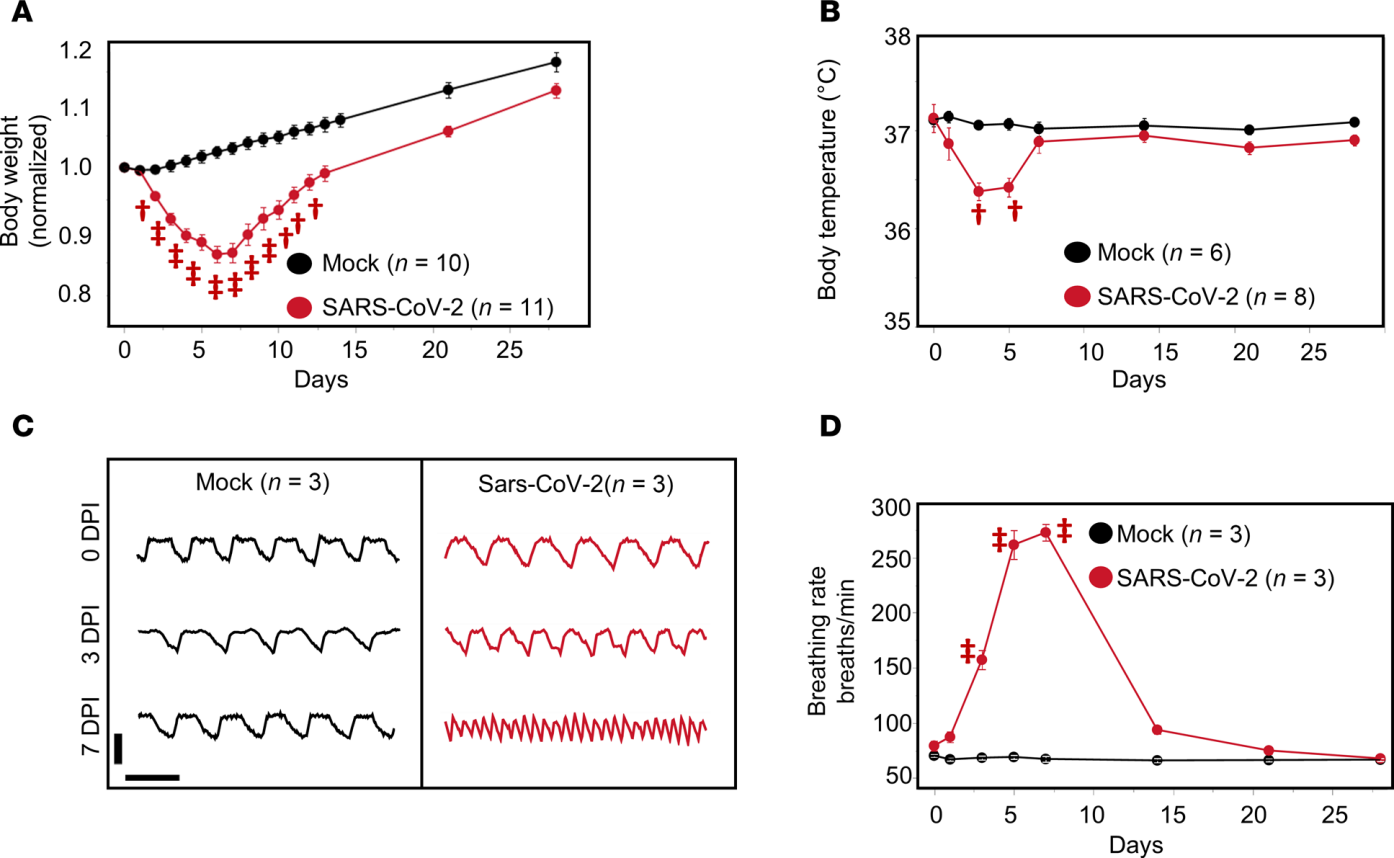
SARS-CoV-2 infection causes weight loss, hypothermia, and tachypnea in the hamster model. (**A**) Body weight decreased after infection, reached a minimum at 6–7 dpi, and then recovered back to normal. (**B**) SARS-CoV-2 infection induced a transient decrease in body temperature peaking at 3 dpi and recovering by 7 dpi. (**C**) Representative traces of subpleural pressure recorded in mock-infected (left) and SARS-CoV-2 (right) –infected hamsters at 0 (upper), 3 (middle), and 7 (lower) dpi. Vertical bar: 10 mmHg; horizontal bar: 1 second. (**D**) Breathing rate increased following SARS-CoV-2 infection, peaked at 7 dpi, and recovered by 14 dpi. ^†^*P* < 0.01; ^‡^*P* < 0.0001.

In humans, systemic inflammatory responses to pathogens often involve either hyper- or hypothermia, tachypnea, and weight loss (38). While the majority (~80%) of patients with COVID-19 present with early fever, hypothermia (39) and weight loss (40) are also associated with poor prognosis. In the hamster COVID-19 model, intranasal inoculation with SARS-CoV-2 (Delta strain) resulted in weight loss, tachypnea, and hypothermia within the first 7 days postinfection (dpi). Consistent with a previous study from our institution (41), weight loss was maximal at 6 dpi, with a 14% ± 1% decrease compared with baseline, followed by reversion to a normal rate of growth by 28 dpi compared with the mock-infected group (Figure 1A).

Dual biopotential/pressure radiotelemetry devices were used to monitor the electrocardiogram (ECG), subpleural pressure, and body temperature simultaneously in freely moving animals. Body temperature decreased after infection, reaching a minimum at 3–5 dpi (37.1 ± 0.1°C mean ± SEM at baseline vs. 36.4 ± 0.1°C at 3 dpi), then recovered to 37.0 ± 0.1°C by 7 dpi (Figure 1B). Tachypnea developed rapidly between 1 and 5 dpi, from 81 ± 6 breaths/min at baseline to 276 ± 18 and 272 ± 11 breaths/min at 5 and 7 dpi, respectively, then recovered to 101 ± 3 breaths/min by day 14 (Figure 1, C and D).

### Triphasic effects of COVID-19 infection on cardiac arrhythmias.

**Figure 2:**
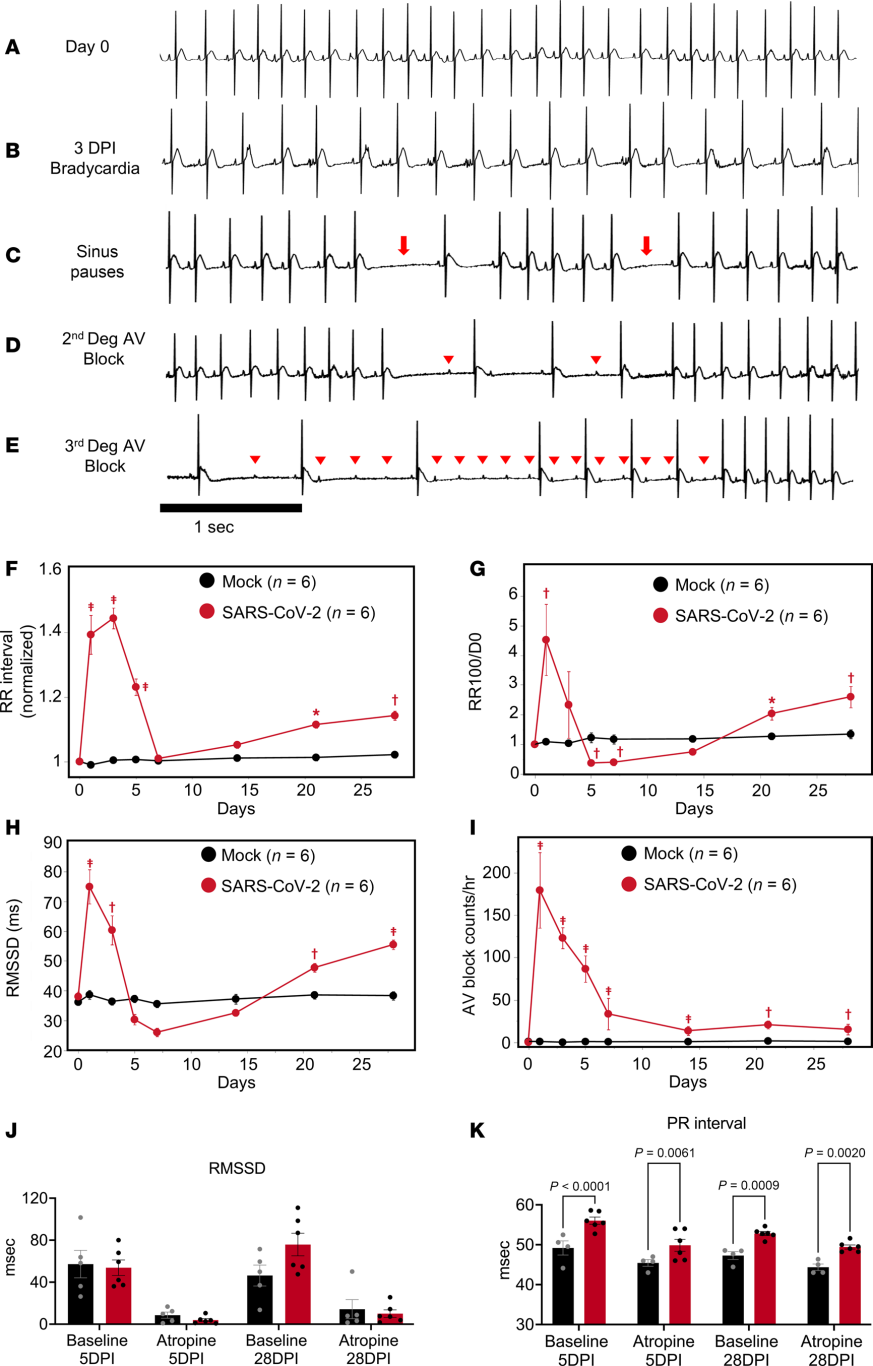
Effects of SARS-CoV-2 infection on cardiac electrophysiology in the hamster model. (**A**–**E**) Representative ECG recording showing normal baseline rhythm (**A**) and arrhythmias induced by SARS-CoV-2 infection, including bradycardia (**B**), sinus pauses (**C**; arrows), second-degree AV block (**D**), and third-degree AV block (**E**). Arrowheads indicate P waves. (**F**–**I**) Analysis of ECGs from 0 to 28 dpi typically showed a triphasic pattern of SARS-CoV-2 effects on cardiac rhythm: an acute peak within 7 dpi; recovery to, or below, baseline; and a long-term effect developing between 7 dpi and 28 dpi. Mean RR interval (normalized to 0 dpi) (**F**), incidence of long sinus pauses (RR > meanRR+100 ms; normalized to 0 dpi) (**G**), and RMSSD (**H**) peaked at 1–3 dpi; returned to levels close to or lower than baseline; and then gradually increased to levels significantly higher than baseline at 21 and 28 dpi. The rate of AV block events (**I**) peaked early and did not return to baseline level, remaining significantly higher than baseline between 7 and 28 dpi. Red symbols denoting *P* values *P < 0.05; ^†^*P* < 0.01; ^‡^*P* < 0.0001 on the figures compare mock- vs. SARS-CoV-2–infected groups. *P* values comparing SARS-CoV-2 data from day 0 through day 28 are available in Supplemental Table 4. (**J**) Atropine decreased RMSSD to a similar extent at 5 dpi and 28 dpi in SARS-CoV-2– (red bars) and mock-infected (black bars) hamsters. (**K**) The increased PR interval in the SARS-CoV-2 group was not abolished by atropine at 5 dpi or at 28 dpi (right). (Two-way ANOVA was performed with α = 0.05.)

ECG analysis revealed that SARS-CoV-2 infection resulted in multiple types of cardiac arrhythmias linked to CCS dysfunction, including bradycardia, sinus pauses, and second- and third-degree atrioventricular (AV) block (Figure 2, A–E). The effects had a triphasic pattern: an early peak at 1–3 dpi, a recovery phase by 7 dpi, and arrhythmia redevelopment that persisted through 28 dpi. As early as 1 day after SARS-CoV-2 infection, marked bradycardia was observed. Compared with baseline, RR interval increased by 39% ± 6% at 1 dpi and peaked at 3 dpi, with an increase of 44% ± 3%, which then reverted to near-baseline levels by 7 dpi (Figure 2F). This was followed by a linear increase in RR interval extending to 4 weeks postinfection that was statistically significant after 21 dpi (11% ± 1%, *P* < 0.001 at 21 dpi and 14% ± 1%, *P* < 0.0001 at 28 dpi) (Figure 2F). The incidence of beat-to-beat pauses longer than the RR interval mean+100 ms (RR100) spiked early at 1 dpi (4.5-fold higher than 0 dpi, *P* < 0.0001), dropped below baseline at 5–7 dpi, and then increased above baseline at 28 dpi (2.6-fold higher than baseline) (Figure 2G).

Evidence of autonomic nervous system dysfunction was also observed but could only partly explain the phenotype. Heart rate variability analysis revealed that the root mean square of the successive RR differences (RMSSD) was significantly higher than baseline at 1 and 3 dpi and lower at 5 and 7 dpi (74.9 ± 5.7 ms at 1 dpi; 60.3 ± 4.9 ms at 3 dpi; 30.4 ± 1.7 ms at 5 dpi; 26.1 ± 1.4 ms at 7 dpi vs. 38.1 ± 1.6 ms at 0 dpi), suggesting increased parasympathetic activity followed by decreased vagal activity during the subacute COVID-19 phase (Figure 2H). RMSSD then increased to levels significantly higher than baseline by 21 and 28 dpi. AV block frequency peaked at 1 dpi (179.50 ± 44.53 vs. 0.19 ± 0.09 at 0 dpi, *P* < 0.0001) and gradually decreased to 33.56 ± 18.54 (*P* < 0.0001 compared with 0 dpi) at 7 dpi but remained elevated through 28 dpi (Figure 2I). Notably, both the bradycardia and AV block events were still present at time points (e.g., 5 dpi) when RMSSD was not significantly different from the mock-infected group, indicating that increased vagal tone alone could not account for the electrophysiological phenotype. To determine whether the CCS was dysfunctional in the SARS-CoV-2–infected hamsters, we performed additional experiments with the cholinergic muscarinic antagonist atropine to block parasympathetic responses at 5 and 28 dpi (Figure 2, J and K). Inhibition of parasympathetic activity by atropine decreased RMSSD to a similar extent in SARS-CoV-2– and mock-infected hamsters, whereas the increased PR interval in the SARS-CoV-2 group was not abolished by atropine. Thus, we conclude that while autonomic dysfunction contributes to SARS-CoV-2–induced arrhythmias, intrinsic CCS dysfunction is also present.

### Immune cell remodeling in the CCS.

**Figure 3:**
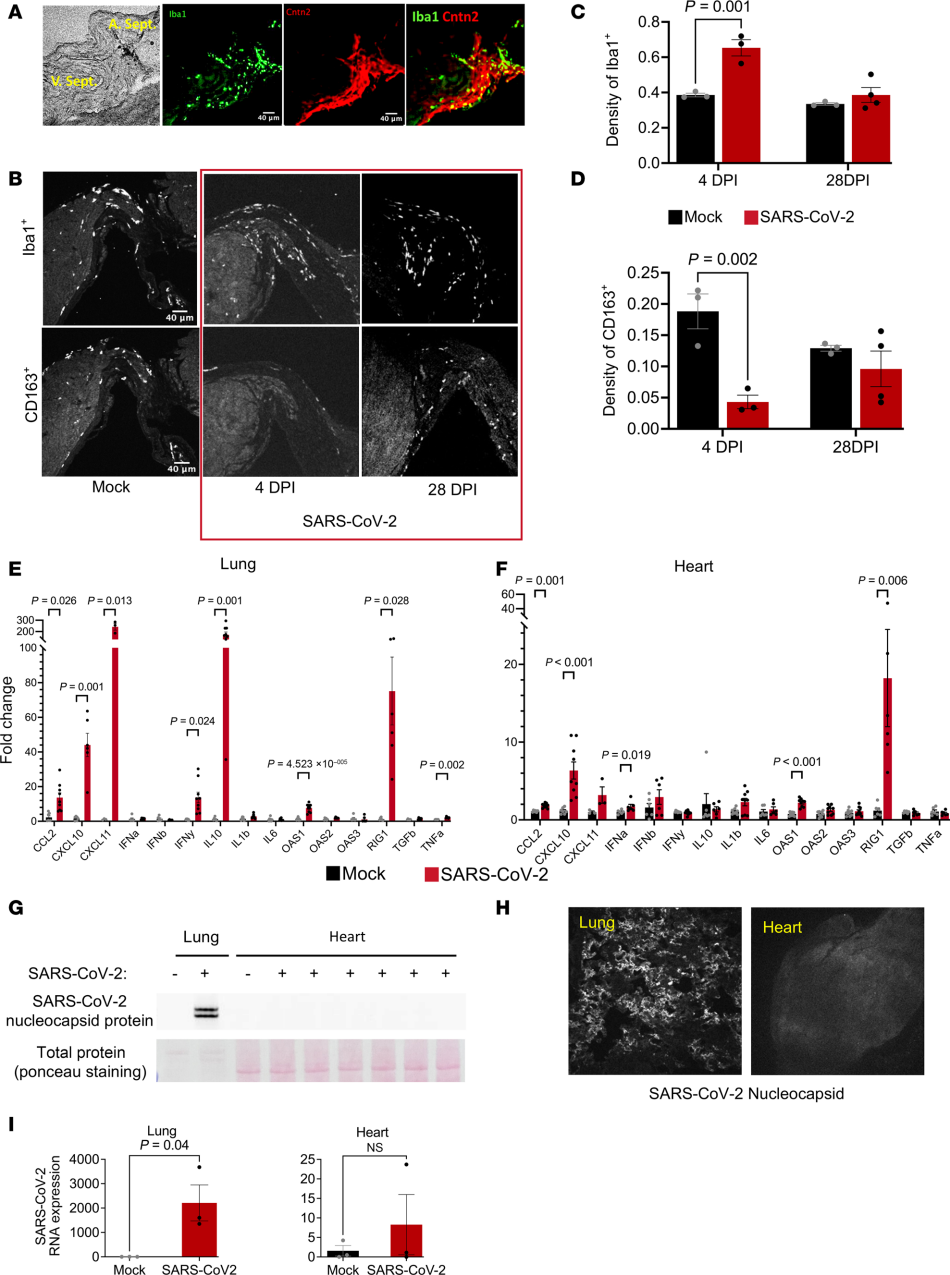
Macrophage remodeling in the CCS. (**A**) Representative images showing the conduction tissue marker contactin 2 (red) and the distribution of Iba1^+^ macrophages (green) in the region. (**B**) Representative images showing Iba1^+^ and CD163^+^ macrophages in the conduction region in mock- or SARS-CoV-2–infected hearts at 4 and 28 dpi. (**C** and **D**) Density of Iba1^+^ cells and CD163^+^ cells in CCS region of the mock- or SARS-CoV-2–infected hearts at 4 and 28 dpi. (**E** and **F**) Gene expression of cytokines and IFN-stimulated genes in lung (**E**) and heart (**F**) at 4 dpi, evaluated with qPCR. Unpaired 2-tailed *t* test was used to test for significance in normally distributed lung data in **E**. Mann-Whitney *U* test was used as the nonparametric significance test in heart data in **F**. (**G** and **H**) SARS-CoV-2 nucleocapsid protein was detected in lung, but not in the heart, by Western blot (**G**) or by immunofluorescence (**H**) at 4 dpi. (**I**) Viral spike protein mRNA was detected in lung, but not in the heart, by qPCR at 4 dpi. Unpaired 2-tailed *t* test.

Previous reports highlighted the important role of colocalized macrophages in the modulation of specialized pacemaker and conduction system cells in the heart. Resident macrophages were shown to facilitate cardiac conduction (42) and enable mitochondrial quality control (43), while recruited macrophages contribute to inflammation and cardiac arrhythmias (44). Hence, we examined whether immune cell remodeling took place in hearts during COVID-19. Tissue slices encompassing conduction system structures at the AV septum (at the level of the His bundle) were positive for the conduction system marker contactin2 (Cntn2) (Figure 3A) (42, 45). The total numbers of macrophages (Iba1^+^) and those expressing CD163^+^, a marker of antiinflammatory or resident macrophages (46), were counted and normalized to tissue pixel area in the AV node/His bundle region at both the acute phase (4 dpi) and postinfection phase (28 dpi) (Figure 3, B–D). In the mock-infected control heart, the relative densities of Iba1^+^ and CD163^+^ macrophages were higher in the AV/bundle region compared with ventricles (Iba1^+^ macrophages: 0.37 ± 0.01 in bundle vs. 0.09 ± 0.01 in ventricles, *P* < 0.0001; CD163^+^ macrophages: 0.16 ± 0.02 in bundle vs. 0.03 ± 0.01 in ventricles, *P* < 0.005; see Supplemental Figure 2; supplemental material available online with this article; https://doi.org/10.1172/jci.insight.193164DS1). Following SARS-CoV-2 infection at 4 dpi, significant macrophage infiltration was observed throughout the heart, and the density of Iba1^+^ macrophages increased by 78% in the AV node/His bundle region of COVID-19 animals compared with mock-infected controls (Figure 3C). Despite the increase in macrophage infiltration, the density of CD163^+^ macrophages decreased by 74% in the AV node/His bundle region. After the acute phase, at 28 dpi, Iba1^+^ macrophage density declined to levels not significantly different from controls (Figure 3C). CD163^+^ macrophage density was also not significantly different from controls at 28 dpi, in part because of a decrease in the mock-infected group (Figure 3D).

### IFN-stimulated gene and cytokine expression in lung and heart.

RT-PCR was used to characterize cytokine expression the lung and the heart. In the lung at 4 dpi, we observed pronounced increases in the antiinflammatory cytokine IL-10 (150-fold), the monocyte chemoattractant CCL2 (12-fold), and IFN-γ (12-fold), as well as downstream IFN-stimulated genes, including the immune cell chemoattractants CXCL11 (241-fold) and CXCL10 (45-fold) (Figure 3E), previously linked to poor outcomes in patients with COVID-19 (47–50). Antiviral innate immune response dsRNA pattern recognition receptors, OAS1 (8-fold) and RIG-I (75-fold), were also elevated in the lung (Figure 3E). Despite the lack of evidence of widespread myocardial SARS-CoV-2 infection (see below), a significant cytokine response, including marked activation of the IFN-stimulated gene response, was observed in the heart at 4 dpi (Figure 3F). Significant increases in RIG-I (18-fold), OAS1 (2-fold), CXCL10 (7-fold), and CCL2 (2-fold) were observed. There were no significant changes in IL-10, TGF-β, or TNF-α in the heart. Although previous reports showed IL-6 elevation with COVID-19 in humans (51), in our study, IL-6 expression was not significantly increased in the lungs or hearts (Figure 3, E and F) of SARS-CoV-2–infected hamsters. Plasma cytokine levels were assessed and significant increases were observed for IFN-γ and monocyte chemoattractant protein-1 after SARS-CoV-2 infection (Supplemental Figure 3).

### Viral protein expression in lung but not heart.

To test whether SARS-CoV-2 was directly infecting the heart, we probed for viral nucleocapsid expression in whole heart and lung homogenates by Western blot or by immunofluorescence in lung or myocardial tissue slices (Figure 3, G and H). Western blot showed no detectable viral nucleocapsid protein in myocardial lysates at 4 dpi, while 2 bands were readily detected in infected lungs (Figure 3G). Widespread nucleocapsid expression was evident in the lung at 4 dpi (Figure 3H) but was absent in the myocardium (Figure 3H). Consistent with protein expression, quantitative measurement with qPCR also revealed significant viral RNA expression in the infected lung at 4 dpi, while no viral RNA was detectable in heart or mock-infected lung (Figure 3I). Although we found no evidence of viral replication in the myocardium, SARS-CoV-2 RNA was present in the serum 4 days postinfection (Supplemental Figure 4), consistent with a previous report in the hamster COVID-19 model (52). These data suggest that the cardiac effects of SARS-CoV-2 infection are an indirect end organ or systemic inflammatory response to pattern recognition receptor activation rather than to direct viral infection and replication in cardiomyocytes.

### Effects of viral dsRNA activation of innate immune response on cardiac electrophysiology in guinea pigs.

**Figure 4:**
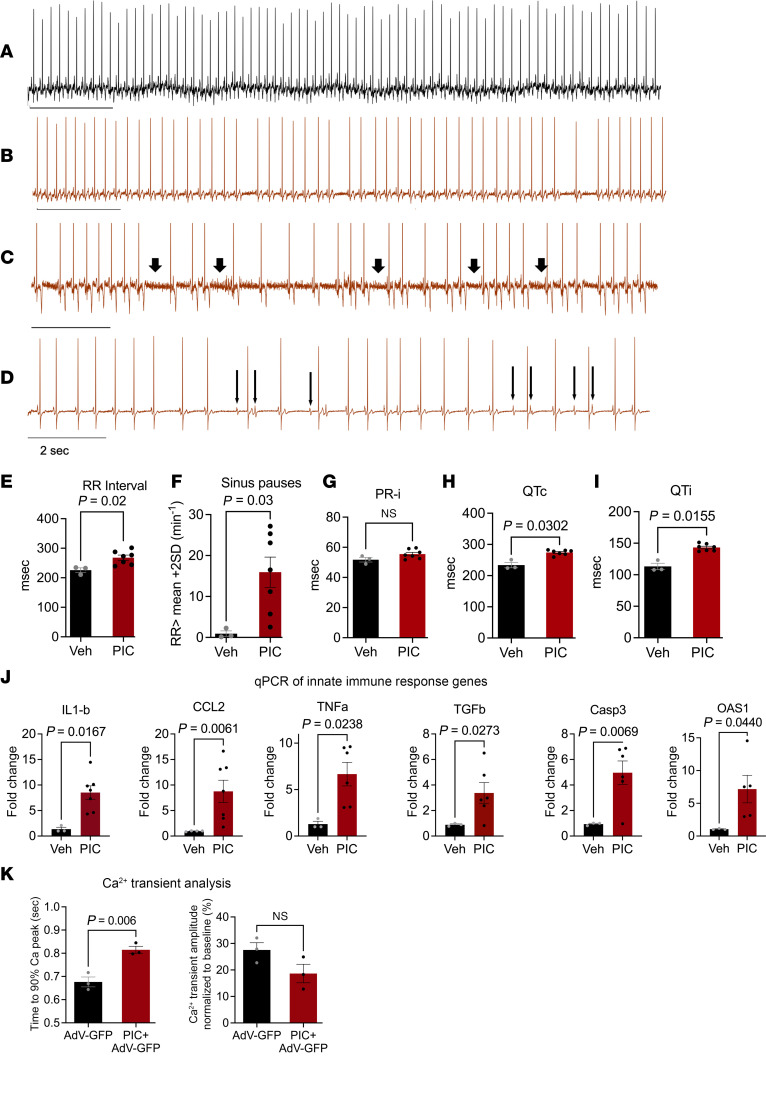
The dsRNA mimetic PIC induces cardiac arrhythmias and innate immune responses in the absence of viral infection in guinea pigs. (**A**–**D**) Representative ECG traces showing regular cardiac rhythm in a vehicle-injected control (**A**) and bradycardia (**B**), sinus pauses (**C**; marked by arrows), and AV block (**D**) after cardiac injection of polyinosinic:polycytidylic acid (PIC) in guinea pigs (arrowheads in **D** indicate abnormal P waves; horizontal bar equals 2 seconds). (**E**–**I**) Summary data showing increase in RR interval (**E**), sinus pauses longer than mean RR+2SD (**F**), corrected QT (**H**), and QT interval (**I**) but no increase in PR interval (**G**) 4 days after myocardial PIC injection in guinea pigs. Welch’s 2-tailed *t* test was done for **B**–**F**. (**J**) Increased expression of innate immune response genes induced by PIC, expressed as fold-change from vehicle-injected controls, was observed. Mann-Whitney test was done for IL-1β, CCL2, and TNF-α. Welch’s 2-tailed *t* test was performed for TGF-β, Casp3, and OAS1. (**K**) Ca^2+^ transient analysis of adult cardiomyocytes isolated from guinea pig hearts injected with either PIC+AdV-GFP or AdV-GFP alone (AdV-GFP was used to identify myocytes near the injection site; unpaired 2-tailed *t* test).

The strong activation of antiviral IFN signaling in the hamster COVID-19 model, in the absence of direct evidence of cardiac viral infection, raised the question of whether the ECG phenotype might be indirectly linked to the innate immune response to circulating viral RNA or myocardial viral infiltrates (19). Hence, we sought to determine if a viral dsRNA mimetic (polyinosinic:polycytidylic acid, PIC) was sufficient to mimic the cardiac electrophysiological phenotype. We utilized naive guinea pigs for a onetime direct myocardial injection of PIC into the left ventricular free wall. This species was used because of the similarity of its electrophysiological profile to humans, including a long action potential plateau, and repolarizing currents dominated by the rapid and slow components of the delayed rectifier potassium channels (53–55), thereby enabling a closer analysis of repolarization. Remarkably, bradycardia, sinus pauses, and AV nodal dysfunction were observed in PIC-treated guinea pigs but not vehicle-injected controls (Figure 4, A–D). Mean RR was significantly increased by PIC treatment compared with controls (225.9 ± 7.7 ms in control vs. 268.0 ± 8.7 ms in PIC, *P* < 0.05) (Figure 4E). The incidence of sinus pauses in the PIC group (RR>RRmean+2SD) increased by 17-fold (15.9 ± 3.7 in PIC vs. 0.9 ± 0.7 in control, *P* < 0.05) (Figure 4F). Significant increases in QT corrected (233.9 ± 8.5 ms in control and 274 ± 3.08 in PIC) and QT interval (113.2 ± 5.07 ms in control and 143.2 ± 2.09 ms in PIC, *P* < 0.05) (Figure 4, H and I) were observed. However, the PR interval was not significantly different between control and PIC treatment (Figure 4G). PIC treatment evoked a cardiac innate immune response, with elevated expression of OAS1, CCL2, TGF-β, IL-1β, TNF-α, and caspase-3 (Casp3) mRNAs (Figure 4J). Further, in vivo myocardial PIC injection altered Ca^2+^ handling in cardiomyocytes isolated from the guinea pig hearts. Hearts injected with PIC+AdV-GFP (an adenoviral vector expressing GFP) were compared with those injected with AdV-GFP alone. The AdV-GFP was used to mark cells near the injection sites. Ca^2+^ transient duration at 90% of decay was increased and Ca^2+^ transient amplitude was decreased in myocytes from PIC-injected hearts (Figure 4K).

### Effects of innate immune activation on hiPSC-CM.

**Figure 5:**
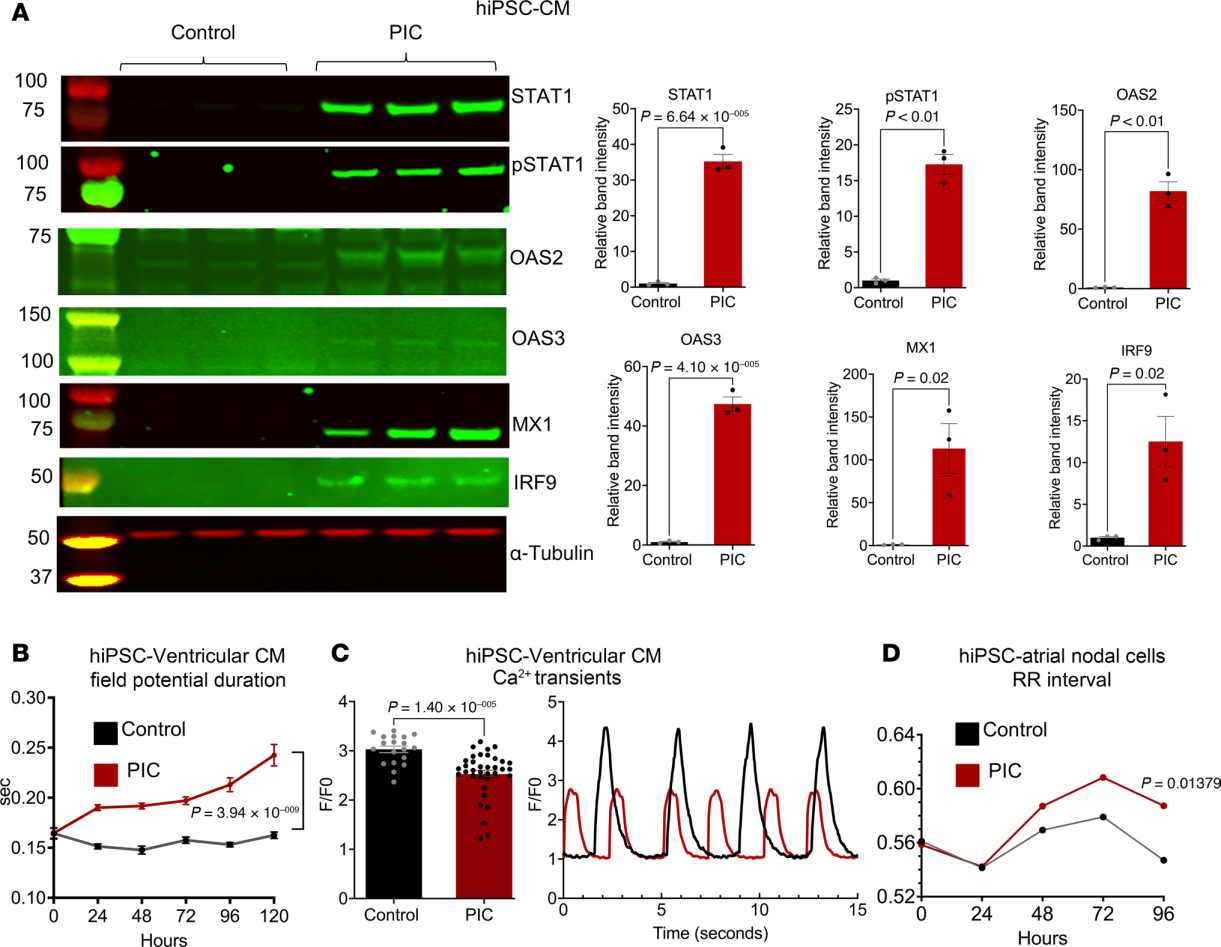
PIC increases expression of antiviral interferon signaling proteins and alters excitation-contraction coupling in human ventricular and sinoatrial nodal myocytes. (**A**) Activation of interferon signaling pathway proteins in hiPSC-CM with PIC treatment (200 µg/mL for 72 hours). Left panel: Western blot; right panels: quantification of proteins after normalizing to total protein. (*n* = 3; unpaired 2-tailed *t* test.) (**B**) Field potential durations in hiPSC ventricular myocytes were significantly increased by 24 hours after PIC treatment (*n* = 11 for control and *n* = 12 for PIC treated; 2-way repeated measures ANOVA with Holm-Šídák’s multiple-comparison test for panels **B** and **D**). Multielectrode array measurements were taken at the indicated times before and after treatment. (**C**) Left: Ca^2+^ transient peak amplitudes were significantly reduced 5 days after PIC treatment in monolayers of hiPSC-CM (data points represent 20–30 replicates from 3 independent experiments; a nested analysis with Mann-Whitney 2-tailed test was used). Right: Ca^2+^ transient recordings in PIC-treated (red trace) and control (black trace) monolayers. (**D**) In hiPSC-CM atrial/sinoatrial nodal cell monolayers, RR intervals significantly increased 96 hours after PIC treatment (*n* = 6,6). IRF3, interferon response factor 3; pIRF3, phosphorylated IRF3; IFN-β1, interferon β1; Stat1, signal transducer and activator of transcription 1; pStat1, phosphorylated Stat1; OAS1, 2′-5′-oligoadenylate synthase 1; MX1, MX dynamin like GTPase 1; IRF9, interferon response factor 9.

PIC treatment was used to examine the effects of activating the viral RNA-dependent innate immune response, independent of viral infection, on IFN signaling, cytokine release, and function. In hiPSC-CM monolayers, which are differentiated toward a ventricular phenotype, PIC activated IFN-stimulated protein expression. Significant increases in MX1, OAS2 and -3 isoforms, and interferon regulatory factor 9 (IRF9) and increases in STAT1 protein expression and phosphorylation were observed (Figure 5A). To examine electrophysiological effects, we performed microelectrode array experiments in hiPSC-CM monolayers and found that field potential duration, a surrogate for action potential duration, was significantly increased as early as 24 hours after PIC treatment (Figure 5B). To determine the effects of dsRNA innate immune activation on cardiac excitation-contraction coupling, we measured Ca^2+^ transients in hiPSC-CM monolayers treated with PIC. Innate immune activation resulted in a significant decrease in Ca^2+^ transient peak amplitude (Figure 5C) with no significant effect on diastolic Ca^2+^ or spontaneous beating rate (Supplemental Figure 5). To assess PIC effects on hiPSC-derived pacemaker cells, we employed an additional retinoic acid treatment protocol to differentiate the hiPSCs toward a sinoatrial node/atrial myocyte phenotype, which induced them to express pacemaker cell-specific markers (Supplemental Figure 6). Microelectrode array analysis of sinoatrial/atrial monolayers showed that PIC significantly increased RR interval at 96 hours (Figure 5D).

### Innate immune activation suppresses mitochondrial respiration in a lung epithelial cell model.

In a human lung epithelial cell line, A549, representing a frontline target of SARS-CoV-2, PIC treatment activated the RNA pattern recognition pathway and the IFN response. IRF3 and STAT1 phosphorylation were increased, together with increases in STAT1, IFN-β1, and OAS1, -2, and -3 protein expression (Supplemental Figure 7A). In addition, RNA degradation was elevated by PIC, consistent with activation of RNase L (Supplemental Figure 7B). Furthermore, we observed that mitochondrial oxidative phosphorylation was markedly suppressed in A549 cells. Both basal and maximal (uncoupled) oxygen consumption rate were decreased, nearly eliminating spare respiratory capacity (Supplemental Figure 7C). The bioenergetic dysfunction after the immune challenge was mediated by excess mitochondrial ROS, as pretreatment with the mitochondrially targeted antioxidant mitoTEMPO (56, 57) enhanced basal, maximal, and spare respiratory capacity to levels even higher than the control group (Supplemental Figure 7C).

### In vitro assessment of JAK/STAT inhibition or mitochondrial ROS scavenging.

**Figure 6:**
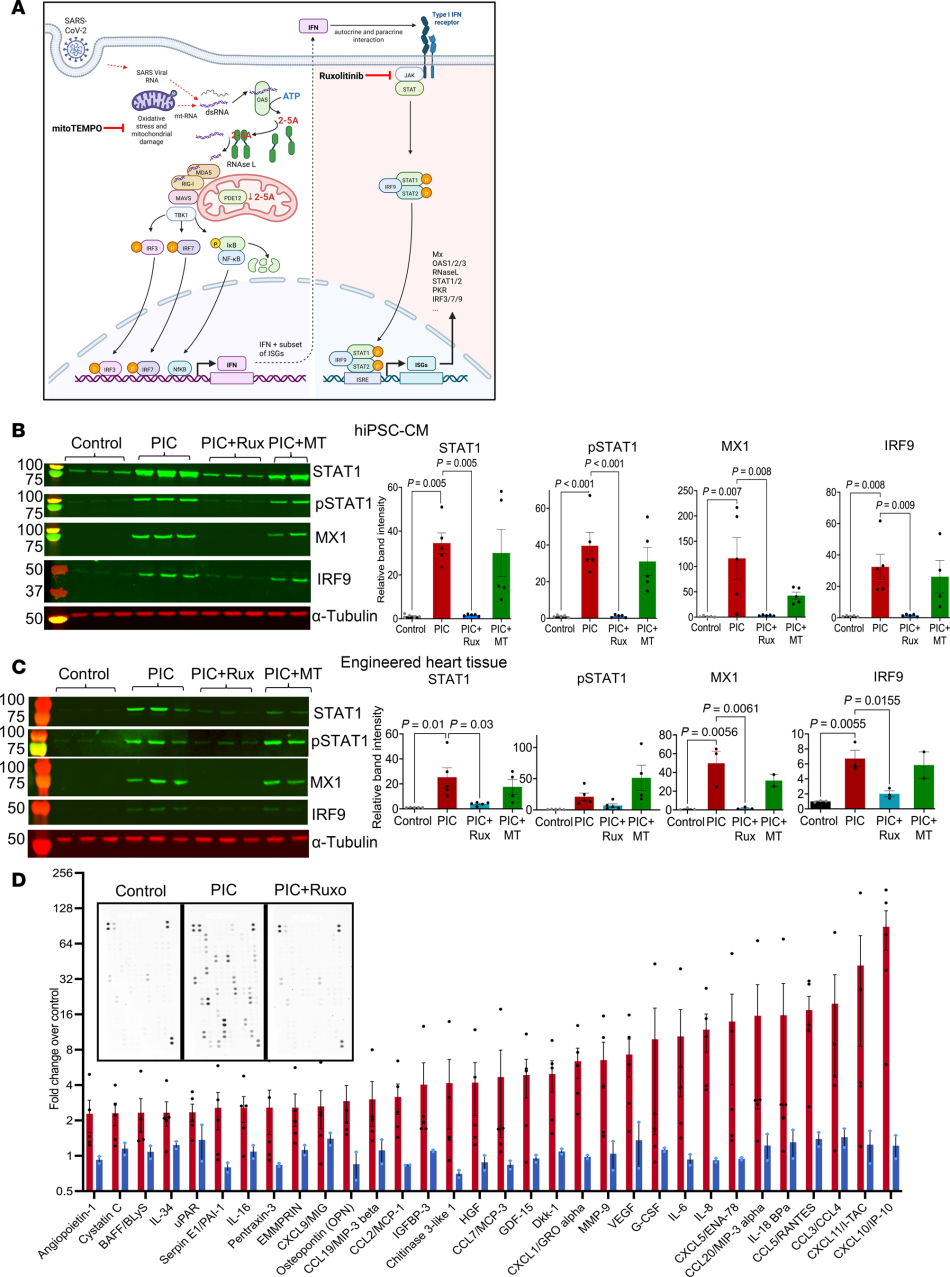
JAK/STAT inhibition, but not mitochondrial antioxidant treatment, suppresses interferon responses in hiPSC-CM. (**A**) Schematic of molecular mechanisms of COVID-19 and dsRNA induced IFN response. Ruxolitinib suppresses IFN-stimulated response and mitoTEMPO suppresses mitochondrial damage and oxidative stress. (Created with BioRender.com.) (**B** and **C**) Inhibition of JAK/STAT signaling with ruxolitinib (Rux) (1 μM) significantly suppressed PIC-mediated induction of the interferon response signaling proteins STAT1, p-STAT1, IRF9, and MX1 in hiPSC-CM monolayers. MitoTEMPO (MT) (1 μM) does not significantly suppress the IFN response. Bar graphs show relative band intensity normalized to total protein loading (1-way ANOVA with Tukey’s multiple-comparison test). Lysates were from 3–5 different hiPSC-CM samples. (**C**) Inhibition of JAK/STAT signaling in hiPSC-derived engineered heart tissues (EHTs) with ruxolitinib, but not mitoTEMPO (MT), decreased the interferon response of signaling proteins STAT1, pSTAT1, IRF9, and MX1. Lysates loaded for the Western blot in **C** were from 3 different EHTs. (**D**) Cytokine secretion into the media induced by PIC, measured using a human Proteome Profiler array (inset shows exemplar blots), was greatly suppressed by Rux treatment in hiPSC-CM.

Ruxolitinib (Ruxo) is a potent antiinflammatory and immunosuppressive agent, clinically used as a JAK1/JAK2 inhibitor, and is commonly prescribed for autoimmune diseases (58, 59). While JAK/STAT inhibitors (e.g., baricitinib or Ruxo) showed some positive outcomes in the treatment of hospitalized patients with COVID-19 (60), the need for additional well-designed randomized trials was emphasized. This has been a challenge because (a) the level of baseline immunity to SARS-CoV-2 changed after vaccines were developed, and (b) the nature of the virus has changed due to mutation and will continue to do so in the future. Hence, the animal model remains a powerful tool to test mechanistic hypotheses. Oxidative stress plays a role in the innate immune response and the cytokine storm, and several antioxidant strategies were tested as potential treatments for complications associated with COVID-19 (61). To target these 2 important nodes (Figure 6A), we tested whether JAK/STAT inhibition with Ruxo or mitochondrially targeted antioxidant therapy (mitoTEMPO) altered the innate immune response in vitro or abrogated COVID-19 effects in vivo.

In hiPSC-CM, JAK/STAT inhibition with Ruxo (1 μM) suppressed IFN-stimulated protein expression (STAT1, p-STAT1, MX1, IRF9) and cytokine responses induced by PIC (Figure 6B). To understand the extent of oxidative stress in the cellular innate immune response, we treated hiPSC-CM with mitoTEMPO (1 μM) and found that it did not significantly suppress the IFN-stimulated protein expression (Figure 6B). We also assessed mitoTEMPO and Ruxo effects on engineered heart tissues (EHTs). Ruxo significantly inhibited PIC-induced increases in STAT1, MX1, and IRF9, while mitoTEMPO treatment had no effect (Figure 6C). Using a human cytokine antibody array (Proteome Profiler, R&D Systems), we found that PIC induced the secretion of a plethora of cytokines into the media from hiPSC-CM, including a >2-fold increase in 36 of the 105 cytokines assayed (Figure 6D and Supplemental Table 3). The most abundant cytokine secreted by the hiPSC-CM was CXCL10 (IP-10), which increased ~90-fold. This finding suggests that myocytes themselves could contribute to the whole-heart increase in CXCL10 expression shown earlier (Figure 3F). Interestingly, CXCL10 was recently linked to the cytokine storm in adults infected with SARS-CoV-2 (48, 50) and is a biomarker of multisystem inflammatory syndrome and left ventricular dysfunction in children with COVID-19 (47). Several other cytokines thought to contribute to the cytokine storm in patients with COVID-19 were secreted by hiPSC-CM after the PIC challenge, including CXCL11 (41.7 ± 33.2-fold; SEM), IL-6 (10.4 ± 7.2-fold) (49), VEGF (7.2 ± 2.6-fold) (62), HGF (6 ± 2.9-fold) (63), CXCL5 (17.3 ± 5.3-fold) (64), IL-8 (11.8 ± 4.2-fold) (65), and CXCL1 (6.3 ± 1.8-fold) (65), potentially representing local mediators of myocardial inflammation, along with CCL5 (17.8 ± 5.5-fold), which was associated with COVID-19 severity (66). Ruxo suppressed cytokine secretion to near control levels (Figure 6D).

### Connexin dysregulation in vitro and in vivo upon activation of the innate immune response.

**Figure 7:**
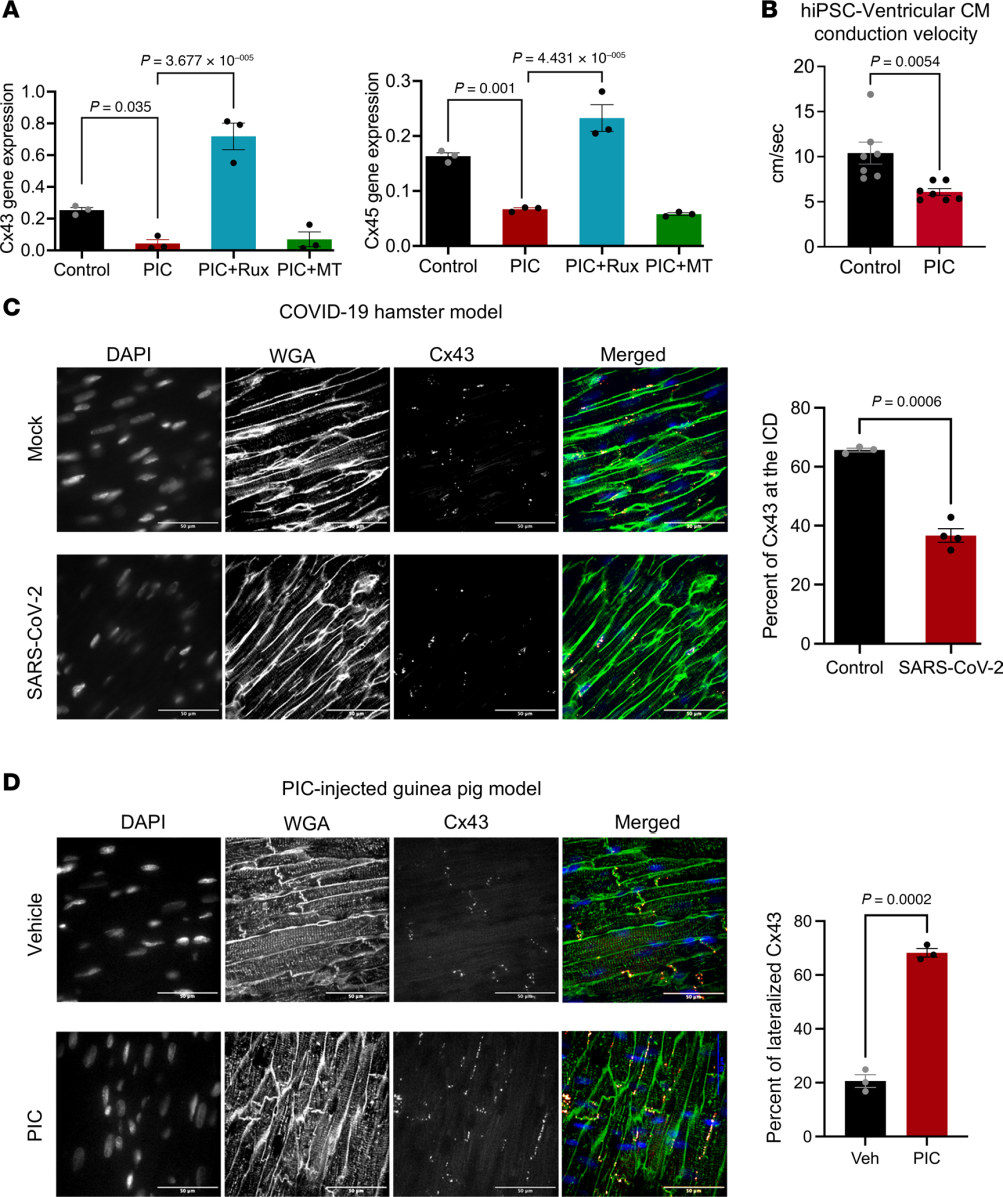
Connexin dysregulation in vitro and in vivo after innate immune activation. (**A**) In hiPSC-CM, Cx43 and Cx45 gene expression was suppressed by PIC (72 hours) and prevented by concomitant ruxolitinib treatment, but not by mitoTEMPO (*n* = 3, 1-way ANOVA with Holm-Šídák’s multiple comparisons test). (**B**) Optical mapping of hiPSC-CM monolayers showed that 4–5 days of PIC treatment significantly decreased conduction velocity (*n* = 7; unpaired nested *t* test). (**C**) Images: Aberrant Cx43 localization in the ventricular myocardium 4 days after SARS-CoV-2 infection in the hamster COVID-19 model. Cx43 was mislocalized away from the intercalated discs (ICD) and toward the nuclear/perinuclear domains. Right-hand panel: Percentage of Cx43 at the ICD. DAPI, nuclear stain; WGA, wheat germ agglutinin membrane marker; Cx43, connexin 43. (**D**) Images: In PIC-injected guinea pig myocardium, Cx43 decreased at the ICD and increased at lateral membranes. Right-hand panel: Percentage of lateralized Cx43 in PIC-injected guinea pig hearts.

The increased arrhythmias and altered ECG parameters in the animal models suggested that cardiac conduction might be altered by innate immune activation. Hence, we tested whether PIC treatment alters connexin expression or conduction velocity in hiPSC-CM. At 72 hours after exposure to PIC, connexin 43 (Cx43) and Cx45 mRNA expression was markedly decreased, which was prevented by Ruxo but not mitoTEMPO (Figure 7A). PIC treatment also significantly decreased conduction velocity measured by high-speed optical mapping of FluoVolt-loaded hiPSC-CM monolayers (Figure 7B). To determine if IL-6 was involved in the reduction in connexin expression induced by PIC, we tested whether the inhibitor tocilizumab (2.5 μg/mL) had any effect, and it did not (Supplemental Figure 8).

Ruxo also significantly suppressed PIC-induced increases in STAT1 and p-STAT1 proteins in A549 cells, but mitoTEMPO did not (Supplemental Figure 9). Overall, the results demonstrated that JAK/STAT inhibition using Ruxo significantly reduces IFN signaling and restores gap junctional gene expression in vitro.

Next, we examined Cx43 distribution in the in vivo animal models. In control (mock-infected) hamster hearts, Cx43 was broadly distributed at the intercalated discs (ICD), lateral membranes, and intracellular locations of the ventricular myocardium, whereas at 4 days after SARS-CoV-2 infection, the percentage of Cx43 at the ICD was significantly decreased, with the remaining Cx43 signal appearing to be localized close to the cell nuclei (Figure 7C). Sterile innate immune activation also resulted in aberrant Cx43 localization. In control (vehicle-injected) guinea pig hearts, Cx43 was predominantly localized to the ICD domains, but PIC-injected hearts displayed prominent relocation of Cx43 to lateral membranes (Figure 7D).

Taken together, our results indicate that innate immune activation disrupts gap junctions in vitro and in vivo and thus could be a key contributor to the increased vulnerability to arrhythmias observed in COVID-19.

### Effects of JAK/STAT inhibition or mitochondrial ROS scavenging on COVID-19–induced pulmonary and cardiac dysfunction.

**Figure 8:**
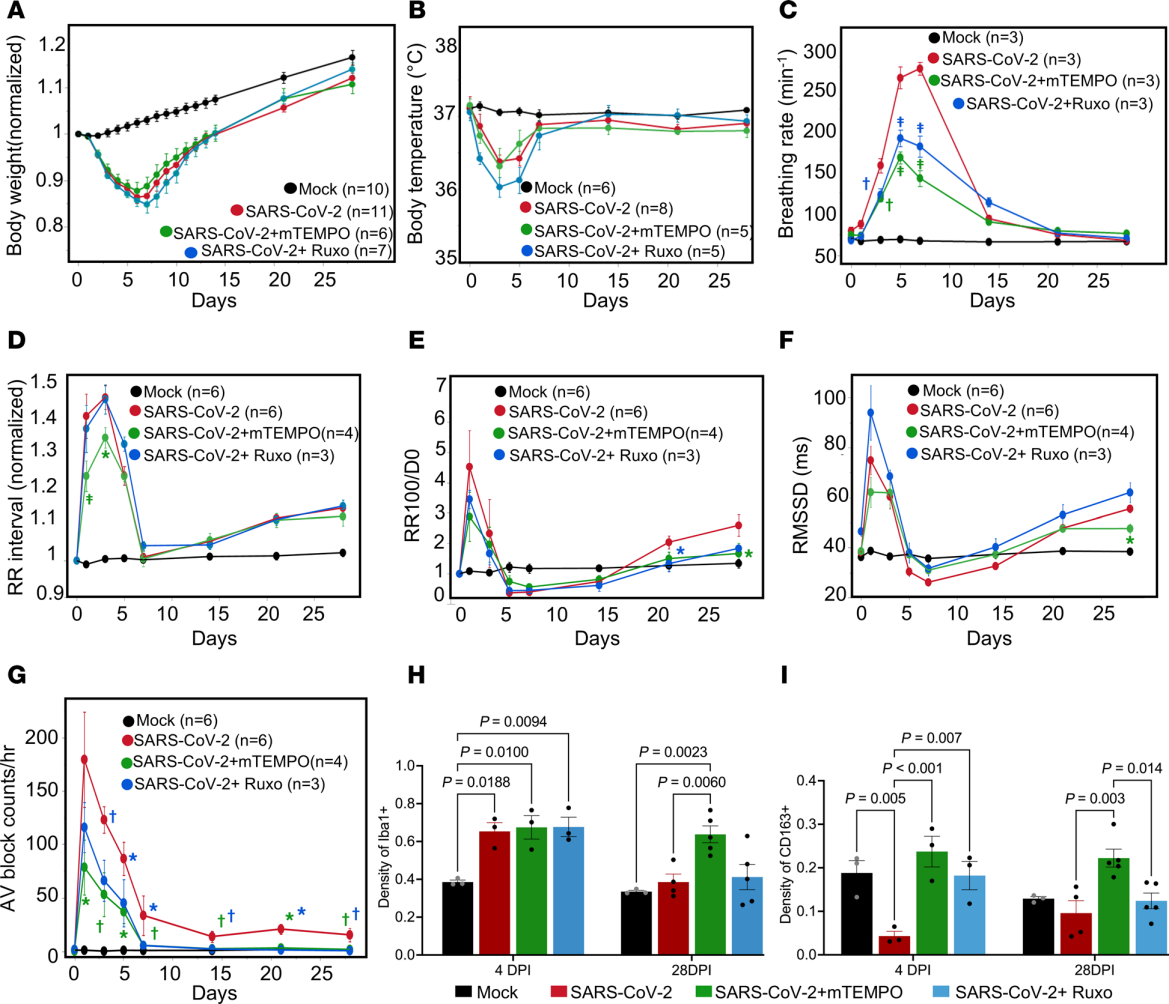
Effects of mitochondrial ROS scavenging or JAK/STAT inhibition on pulmonary and cardiac sequelae of COVID-19. (**A** and **B**) COVID-19–induced changes in body weight (**A**) or temperature (**B**) were not significantly altered by mitoTEMPO (mTEMPO) or ruxolitinib (Ruxo) treatments. (**C**) SARS-CoV-2–induced tachypnea was significantly attenuated by both mTEMPO and Ruxo treatments. ^†^*P* < 0.005, ^‡^*P* < 0.0001. (**D**) mTEMPO, but not Ruxo, attenuated the increase in RR interval at 1 and 3 dpi. After returning to baseline level at 7 dpi, both treatments showed no impact on the redeveloped bradycardia. *P < 0.05, ^‡^*P* < 0.0001. (**E** and **F**) The early spike in sinus pauses (**E**) and RMSSD (**F**) at 1 dpi were not significantly decreased by either treatment. Both Ruxo and mTEMPO attenuated the late effects of SARS-CoV-2 infection on sinus pauses at 21 or 28 dpi, respectively (*P < 0.05). Only mTEMPO significantly suppressed RMSSD at 28 dpi (*P < 0.05). (**G**) Both treatments significantly attenuated the incidence of AV block 1–7 dpi and abrogated the sustained increase in AV block at 14–28 dpi. (*P < 0.05, ^†^*P* < 0.01; compared with SARS-CoV-2 alone, blue symbols are Ruxo treatment, green symbols are mTEMPO treatment; see Supplemental Table 4 for additional within group comparisons with 0 dpi.) (**H**) Iba1^+^ macrophage density increased in the CCS region in all SARS-CoV-2–infected groups, regardless of treatment at 4 dpi, and returned to baseline level at 28 dpi, except in the mTEMPO-treated group). (**I**) Treatment with either mTEMPO or Ruxo prevented the decrease in CD163^+^ macrophage density in the conduction region at 4 dpi. Two-way mixed-effect ANOVA was performed for **H** and **I**. We analyzed 3–11 hamsters per group per parameter. *n* = 3 for groups of 4 dpi and *n* = 4 for groups of 28 dpi. Each heart has 3 repeats. Ruxo and mitoTEMPO treatment data are superimposed on that of mock- and untreated SARS-CoV-2–infected groups reproduced from Figures 1 and 2.

The effects of activation of dsRNA-triggered innate immune signaling on hiPSC-CM function provided motivation to test the therapeutic potential of systemic inhibition of JAK/STAT signaling in the COVID-19 hamster model. Ruxo is one of several clinically utilized JAK/STAT inhibitors (specifically, a JAK1/JAK2 inhibitor) that are potent antiinflammatory and immunosuppressive agents commonly prescribed for autoimmune disease (58, 59). A clinical trial for its use in COVID-19 was initiated, but prematurely truncated by the sponsor, due to low statistical power (67) and FDA approval of the alternative JAK/STAT inhibitor baricitinib for combination therapy, which reduced mortality in hospitalized patients with COVID-19 (68). Oxidative stress also contributes to the innate immune response and the cytokine storm, so antioxidant interventions have been proposed as potential treatments for COVID-19 complications (69–71). To assess the impact of inhibiting JAK/STAT or mitochondrial ROS on pulmonary and cardiac functional parameters in the hamster COVID-19 model, we implanted osmotic pumps for chronic intraperitoneal delivery of Ruxo (2 mg/kg/d) or mitoTEMPO (1.1 mg/kg/d) 4 days prior to SARS-CoV-2 infection and continued until 10 dpi, covering the acute phase of infection. Pulmonary function and ECG analyses were carried out as described above. The results for the 2 treatments are shown (Figure 8) superimposed on the data for the untreated SARS-CoV-2–infected group (replotted from Figures 1 and 2).

Ruxo or mitoTEMPO treatments had minimal effect on the decreases in body weight or body temperature observed after SARS-CoV-2 infection (Figure 8, A and B); however, both treatments significantly inhibited the tachypnea associated with COVID-19 (Figure 8C). Ruxo treatment decreased the peak breathing rate by 27% and 34% at 5 and 7 dpi, respectively, compared with SARS-CoV-2 infection alone. MitoTEMPO treatment decreased the peak breathing rate by 36% and 48% at 5 and 7 dpi, compared with untreated infected animals. MitoTEMPO treatment was more effective than Ruxo treatment at suppressing tachypnea, and the recovery to baseline was faster (Figure 8C). Interestingly, mitoTEMPO treatment improved the cardiac electrophysiological phenotype, blunting the increase in RR interval over the first 7 dpi (Figure 8D). Although mitoTEMPO was only administered during the acute phase, its effect extended to postinfection phase. The prolonged response of RR100 and RMSSD to SARS-CoV-2 infection was suppressed, and there was no significant difference in RR100 or RMSSD at 21 or 28 dpi, as compared with 0 dpi (Figure 8, E and F). Ruxo had no significant effect on sinus bradycardia and autonomic dysfunction associated with COVID-19; however, both mitoTEMPO and Ruxo prevented AV block in the postinfection phase from 7–28 dpi, where the incidence of AV block remained higher than baseline in the group with SARS-CoV-2 infection alone (Figure 8G). We next examined macrophage densities and found that the increase in Iba1^+^ macrophages in the CCS in the COVID-19 group at 4 dpi was not prevented by either of the treatments (Figure 8H); however, both treatments prevented the early decline in CD163^+^ macrophages (resident or M2-type macrophages) (Figure 8I). At 28 dpi, Iba1^+^ and CD613^+^ macrophage counts reverted to the levels of uninfected controls in all the SARS-CoV-2 groups except the mitoTEMPO treated.

## Discussion

This study provides a comprehensive time course and mechanistic investigation of the short- and long-term cardiac arrhythmias induced by COVID-19 in an animal model susceptible to human SARS-CoV-2. The main findings are that (a) COVID-19 causes significant CCS dysfunction, including acute bradycardia, sinus node dysfunction, and AV nodal block, following a triphasic course of transient arrhythmias during peak infection, partial recovery, and late recurrence; (b) dsRNA-triggered innate immune activation reproduces this phenotype without viral infection, altering cytokine release, connexin expression, and excitation-contraction coupling in human cardiomyocytes; (c) macrophage phenotype remodeling occurs in the CCS and ventricles; (d) aberrant myocardial connexin localization follows infection or sterile immune activation; and (e) blocking IFN signaling or mitochondrial oxidative stress mitigates the pulmonary and cardiac electrophysiological effects of COVID-19.

### Cardiovascular consequences of COVID-19.

During the COVID-19 pandemic, the risk of new-onset cardiometabolic disease increased dramatically after SARS-CoV-2 infection. A case-control study of more than 400,000 patient medical records in the UK health system showed large increases in relative risk of diabetes, thrombosis, heart failure, and cardiac arrhythmias that spiked early but persisted for months after the index infection (1). Similarly, a worldwide survey reported that arrhythmias occurred in ~18% of patients with COVID-19 (72), with the majority developing atrial arrhythmias (fibrillation or flutter), ~23% developing bradyarrhythmia (sinus pauses or AV block), and ~20% showing ventricular tachyarrhythmias or fibrillation. A high incidence of arrhythmias was also reported in patients with COVID-19 admitted to the intensive care unit, varying between approximately 17% (6, 73) and 44% (74, 75), with many patients displaying AV block and bradycardia (76–79), associated with worse prognosis (78). Arrhythmias involving impaired CCS activity are also significantly increased in patients suffering from long COVID syndrome (also known as post-acute sequelae of COVID-19, or PASC). These include chronotropic incompetence, increased heart rate variability, and postural orthostatic tachycardia syndrome, which points to long-term dysautonomia or impaired function of the CCS (80).

Since widespread vaccination became available, it has been challenging to unravel the lasting impact of COVID-19 on cardiovascular complications at the population level. A high-dimensional characterization of PASC in US veterans showed that incident adverse cardiac outcomes, including arrhythmias, increased markedly at 6 months after infection, compared with uninfected individuals, with the excess burden highest among those previously hospitalized or in the ICU during their infection (13). Because there are no accepted treatments to forestall persistent COVID-19–induced pathologies (14), it remains critically important to elucidate the mechanisms involved, given the ongoing evolution of SARS-CoV-2, the inability of the vaccines to prevent new infections, and the ongoing potential of SARS-CoV-2 or other respiratory viruses to induce CCS dysfunction (81).

In the context of these human studies, our results show that SARS-CoV-2 infection in hamsters is a robust model to study the bradyarrhythmias, AV block events, and CCS dysfunction associated with COVID-19. Although prominent in humans with COVID-19 (8), spontaneous atrial tachyarrhythmias were not generally observed in this model. Two factors might account for this. First, spontaneous reentrant arrhythmias (e.g., atrial fibrillation) are rarely observed in small animals owing to the small mass and conduction path length of the heart. Thus, most studies utilize programmed stimulation to examine atrial arrhythmia inducibility (82). Second, age is a major cofactor associated with atrial fibrillation in the general population and in patients with COVID-19 (8). Our young adult hamsters lack increased fibrosis and other comorbidities of age that predispose to atrial fibrillation. Nevertheless, the ECG changes that we observed indicate altered excitability and conduction, which could provide a substrate for more complex atrial or ventricular tachyarrhythmias under stress or in the presence of comorbidities. We showed that the hamster model displays long-term effects on sympathovagal balance, which is disrupted in long COVID syndrome (83); however, changes in autonomic tone could not fully account for the observed CCS dysfunction. Increased parasympathetic tone (indicated by RMSSD) was evident early but dropped below baseline in the subacute phase (5–15 dpi), only to reemerge by 1 month after infection. Additional studies are warranted to decipher the relative contributions of intrinsic CCS remodeling versus extrinsic changes in autonomic function in the phenotype we observed.

### Cardiac innate immune responses contribute to cardiac arrhythmias.

The mechanisms underlying the increased arrhythmia incidence in COVID-19 are unknown. Early studies of autopsy samples suggested that replicative SARS-CoV-2 RNA is present in the hearts of patients with COVID-19 (17, 18). This view has given way to the consensus that viral RNA may be present (19), but little direct myocardial infection occurs (25–28). Nevertheless, evidence of cardiac inflammation is widely reported (21, 84–87), supporting a systemic cytokine storm as a possible mechanism (21, 88–90). Consistent with many human studies, we found no evidence of viral protein expression in hearts of hamsters after infection with SARS-CoV-2. This differs from a previous study reporting approximately 10%–15% of myocytes with positive antibody signals for viral spike or N protein (91). The reason for this discrepancy is unclear but could be related to SARS-CoV-2 strain or titer differences. Regardless, we show here that the cardiac arrhythmias, cytokine responses, gap junctional dysregulation, and macrophage infiltration occur in the absence of cardiac viral protein expression and can be mimicked by direct activation of the innate immune response with dsRNA. The opposite changes in the numbers of Iba1^+^ and CD163^+^ macrophages in the AV nodal region early after infection, paralleling CCS dysfunction, are particularly interesting. Tissue-resident macrophages highly express CD163 (46) and can influence cardiac conduction by direct electrical coupling with nodal myocytes (42). They also broadly participate in myocyte mitochondrial quality control by removing damaged mitochondria through an exopher mechanism (43). In contrast, monocyte-recruited inflammatory macrophages can elicit atrial fibrillation (44). We propose that the observed CCS remodeling and altered local crosstalk between myocytes and immune cells contributes to the impaired CCS function. We detected a plethora of chemoattractant cytokines and growth factors released from human cardiomyocytes after activation of the innate immune response. These could act in an autocrine or paracrine manner to alter myocyte function, recruit additional immune cells, and release a different complement of factors, underlining the complexity of the extracellular milieu. Indeed, the connexin dysregulation observed here is likely to be driven by cytokines locally released from the cardiomyocytes and/or recruited macrophages upon IFN pathway activation, as it was sensitive to JAK/STAT inhibition. Altered gap junctional expression or localization has been noted in other models of viral infection, such as adenoviral (92) or Coxsackie virus (93) infection, which can cause arrhythmias and myocarditis in humans. Additional investigation is needed to discern which cytokine/receptor pathways underlie the altered connexin regulation in COVID-19, but our findings implicate pattern recognition receptors as a key target for therapeutic intervention.

### Cytokine profiles.

Increased expression of IFN-stimulated cytokines was present in both lung and heart tissues of SARS-CoV-2–infected hamsters. Several of these have been associated with severity of disease in humans with COVID-19 or other arrhythmogenic diseases. For example, increases in CCL2 and the receptor CCR2 were observed in a murine arrhythmogenic model, along with other inflammatory markers (94), and the CXCL12/CXCR4 axis was identified as a key mediator of atrial fibrillation (95). In the latter case, CXCL12 was upregulated, and treatment with a CXCR4 (receptor for CXCL12) antagonist reduced inflammatory cells and markers in the atrial region and suppressed atrial fibrillation. CXCL10 and CXCL11 have been implicated in the cytokine storm during SARS-CoV-2 infection (47, 48, 50), but they are also recognized as inflammatory mediators in ischemic heart disease and heart failure (96, 97). Elevated IL-6 was associated with bradycardia (98), conduction abnormalities, significant QT prolongation (99), and atrial fibrillation (8, 51, 100, 101) in patients with COVID-19. Among the many IFN-stimulated genes that were upregulated in the hamster COVID-19 model, we did not detect a significant increase in IL-6 expression in the lung, heart, or serum. Notably, plasma IL-6 is mainly elevated in severe cases of COVID-19 in humans, that is, hospitalized patients with compromised respiration and elevated serum C-reactive protein (102), and the hamster model has a much milder phenotype and no mortality. Moreover, in human studies, the beneficial effect of the IL-6 antagonist tocilizumab (odds ratio [OR] 0.83) was absent unless patients had severe symptoms and concomitant glucocorticoid therapy (OR 1.06) and was not present for a different IL-6 receptor antagonist, sarilumab (OR 1.08), confounding the conclusion that IL-6 was critically involved (103).

### dsRNA activation of innate immune responses in the absence of virus.

Interestingly, direct injection of guinea pig hearts with the dsRNA mimetic PIC resulted in arrhythmias that were similar to those observed in the COVID-19 hamsters, i.e., bradycardia, long sinus pauses, and AV nodal block. PIC also increased expression of innate immune response genes, supporting the hypothesis that pattern recognition receptor activation is sufficient to induce the in vivo electrophysiological phenotype of COVID-19. We found that even highly purified (98% enriched) hiPSC-CM monolayers released an abundance of cytokines and growth factors, indicating that myocytes themselves can contribute to the antiviral response, as many of the same ones are also observed in COVID-19. For example, CXCL10 and CXCL11, which are upregulated in patients with COVID-19 (48, 50), were markedly increased in the medium after PIC activation in hiPSC-CM, together with more than 20 other cytokines. PIC also impacted adult guinea pig and hiPSC cardiomyocyte function, blunting cytosolic Ca^2+^ transients and decay kinetics and prolonging repolarization. In hiPSC-CM monolayers, conduction velocity decreased after PIC treatment, consistent with a JAK/STAT-sensitive decrease in connexin expression. Representative of the pacemaker cell phenotype, we also found that RR interval increased in hiPSC-CM–derived sinoatrial nodal/atrial cell monolayers, strengthening the links between innate immune activation and CCS remodeling. Similarly, depressed Ca^2+^ transient amplitude and increased spontaneous Ca^2+^ release events were previously reported in hiPSC-CM and rat ventricular myocytes exposed to serum from COVID-19–infected patients (104), possibly implicating cytokines as mediators of rhythm disturbances that could translate to higher organ-level cardiac arrhythmias. Further studies will be needed to discern which cytokine-receptor combinations are critical to the electrical remodeling associated with COVID-19.

### Therapeutic implications.

The expression of RIG-I, OAS1, and OAS2 genes was significantly increased in the lungs and hearts of SARS-CoV-2–infected hamsters. The 2′,5′-oligoadenylate (2-5A) synthase (OAS) family of proteins are key upstream pattern recognition receptors in the antiviral IFN response. Notably, a gene variant in the OAS1/2/3 gene locus is associated with SARS-CoV-2 susceptibility and alters the antiviral response (105–107). Downstream of OAS, the dsRNA sensors RIG-I and MDA5, together with the mitochondrial antiviral signaling protein, are localized in a complex on the outer mitochondrial membrane (108) that transduces the signal to activate early IFN response genes. Interestingly, OAS is overexpressed in postmortem hearts of patients who died of COVID-19, along with alterations of mitochondrial genes, even though no traces of viral gene expression are present, indicative of a strong cardiac innate immune response and perturbation of mitochondrial energetics (26). Mitochondrial impairment and oxidative stress are recognized intermediaries of the inflammatory response to viral infections (109), and increased levels of circulating mtDNA are correlated with severity and mortality in COVID-19 (110). Mitochondrial dysfunction itself could alter the antiviral IFN response by modifying 2-5A levels (111) or by triggering the release of mtDNA into the cytoplasm to activate the cGAS/STING pathway (109, 112), which was shown to contribute to endothelial cell damage during SARS-CoV-2 infection (113, 114).

Given the distinct inflammatory cascade signature promoted by SARS-CoV-2 and the induction of IFN-stimulated gene expression, we targeted 2 potential mechanisms of action: inhibition of JAK/STAT signaling (with Ruxo) and mitochondrial ROS (with mitoTEMPO). Ruxo is FDA approved to treat certain conditions, primarily myelofibrosis, polycythemia vera, and acute graft-versus-host disease. A clinical trial for therapeutic potential of Ruxo for COVID-19 yielded promising results of reduced mortality and improved secondary outcomes, but the study was terminated because of lack of statistical power (67). Other JAK inhibitors have also been used, such as baricitinib, which showed promising preliminary results (115). MitoTEMPO has been used in preclinical models to mitigate ROS-induced adverse cardiac effects of heart failure, diabetes, or aging. Treatment with either Ruxo or mitoTEMPO markedly blunted the tachypnea associated with SARS-CoV-2 infection, with the latter being somewhat more effective. MitoTEMPO, but not Ruxo, also suppressed the early transient bradycardia after infection, as well as the long sinus pauses at 28 dpi. Interestingly, either Ruxo or mitoTEMPO eliminated the persistent AV block events present at 15–28 dpi in COVID-19 hamsters. Limited information is available about the efficacy of antioxidant therapies in COVID-19, but in vitro work showed that mitoquinol or N-Acetyl cysteine, which enhance antioxidant defenses, inhibited viral replication in monocytes infected with SARS-CoV-2 (116). In 2 small clinical studies, treatment with N-Acetyl cysteine reduced the clinical complications and mortality of patients with COVID-19.

### Limitations.

While PIC has been used as a valuable tool for activating antiviral innate immune signaling, it does not fully recapitulate the complexity of viral infections, including viral replication, tissue tropism, interactions of viral proteins, and the participation of adaptive immunity. These limitations should be considered when interpreting its effects on cardiac function. In our study, innate immune activation by PIC in guinea pigs resulted in both sinus node dysfunction and QTc prolongation, as well as altered calcium handling in isolated myocytes, reflecting a ventricular phenotype in addition to the arrhythmias associated with the CCS. Such changes in ventricular repolarization are much easier to detect in guinea pigs, given their human-like long action potential phenotype, as compared with hamsters. However, our studies were limited by the inability to infect guinea pigs with human SARS-CoV-2 and by biosafety level 3 laboratory restrictions that prevented us from cell or organ isolation experiments from hamsters infected with SARS-CoV-2.

Further, while hiPSC-CM provide a human-relevant model for understanding innate immune signaling and the resulting effects on cardiac cellular electrophysiology, it is known that hiPSC-CM are immature. In addition, the atrial/nodal cell differentiation protocol yields a mixed population of atrial and nodal cells, with a predominance of cells expressing atrial nodal markers. Although this population is more representative of cardiac pacemaker tissue, further purification to obtain a pure sinoatrial nodal phenotype is challenging. Therefore, these in vitro results should be interpreted with caution and may not fully recapitulate adult human cardiac physiology.

### Perspectives.

The present findings are the first to our knowledge to describe the time course and mechanisms of cardiac arrhythmias associated with COVID-19 in an animal model that reproduces a subset of those reported in human studies. The observed bradyarrhythmias and nodal dysfunction point to both acute and chronic remodeling of the CCS, caused by activation of IFN signaling and mitochondrial dysfunction. Intervening in these pathways prevented the adverse cardiac and pulmonary effects, but additional investigation will be needed to determine the extent of permanent CCS remodeling after SARS-CoV-2 infection and if this provides the substrate for known electrophysiological abnormalities associated with long COVID syndrome.

## Methods

### Sex as a biological variable.

This study investigated the mechanisms of COVID-19–induced arrhythmias using an established Syrian Golden Hamster model that mirrors the pulmonary tissue damage and inflammation seen in humans (41). Only male hamsters were included in this study because a prior study showed that females exhibited a milder infection response (41).

### Study approval.

The animal procedures in this study were approved by the Institutional Animal Care and Use Committee of Johns Hopkins University (JHU), in accordance with the National Research Council’s *Guide for the Care and Use of Laboratory Animals* (eighth edition). The Johns Hopkins animal care and use program is accredited by the Association for Assessment and Accreditation of Laboratory Animal Care International. All hiPSC experiments were carried out on existing cell lines with the approval of the JHU Institutional Stem Cell Research Oversight Committee.

### Data availability.

Values for graphs in the figures and additional statistical comparisons for the telemetry parameters are provided in the Supporting Data Values file. Detailed methods are included in the Supplemental Methods file.

## Author contributions

DA, TL, and BOR planned, designed, and coordinated experiments and analysis. DA, TL, MNK, JC, MP, AT, BK, BH, JC, KP, and AS performed experiments or analyzed data. TL, MC, AP, and JV performed or supervised animal experiments. MJR, BLL, and DBF performed or analyzed serum cytokine levels. DA, MNK, DHK, and HCC performed or analyzed microelectrode array analysis. DA, TL, and BOR wrote the manuscript. Order of co–first authors was determined alphabetically.

## Funding support

- This work is the result of NIH funding, in whole or in part, and is subject to the NIH Public Access Policy. Through acceptance of this federal funding, the NIH has been given a right to make the work publicly available in PubMed Central.
- American Heart Association Grants 965158 and 25TPA1478354 (BOR).
- Maryland Stem Cell Research Foundation grant MSCRFL-6005 (BOR).
- National Institutes of Health Training Grant T32HL007227 (DA).
- National Institutes of Health Grant R01HL164936 (DHK).
- National Institutes of Health Grant R01HL156947 (DHK).
- National Institutes of Health Grant UH3 TR003271 (DHK).

## Supplementary Material

Supplemental data

Unedited blot and gel images

Supporting data values

## Version 1 12/22/2025

Electronic publication
