# Supplementary material for: Innate immune activation and mitochondrial ROS induce acute and persistent cardiac conduction system dysfunction after COVID-19
Source: JCI Insight. 2025 Dec 22;10(24):e193164. doi: 10.1172/jci.insight.193164 (PMC12890510; doi:10.1172/jci.insight.193164)
Supplement: Supplemental data [file jciinsight-10-193164-s035.pdf]

Extended Methods

Animal models and study design

Sample size was based on results from previous studies (1–3) and space availability in the BSL-3 lab. 5-11 hamsters (8-10 weeks old, Inotiv, Inc.) per group were included, and one hamster was excluded due to a telemetry-implant surgery-related death. Hamsters were divided into four groups: Mock-infected, SARS-CoV-2, SARS-CoV-2+Ruxo, and SARS-CoV-2+mitoTEMPO. To test whether sterile activation of an innate immune response was sufficient to induce arrhythmias in the absence of viral infection, we used direct cardiac injection of PIC in guinea pigs, as the electrophysiological characteristics of this species resembles humans. 3 male guinea pigs were used as controls, and 7 male guinea pigs were injected with PIC. For hiPSC-CM studies, data were collected from at least 3 different batches of differentiated cells.

SARS-CoV-2 infection experiments in hamsters were performed in a biosafety level 3 facility at Johns Hopkins Research Animal Resources, in compliance with the established ethical guidelines. 10^5 TCID50 (50% tissue culture infectious dose)/ml of SARS-CoV-2/Delta variant (SARS-CoV-2/USA/MD-HP05660/2021; GIAID accession number EPI_ISL_2331507) in 100 µl Dulbecco's modified Eagle medium (DMEM) was intranasally administered (50 µl per nare) to male hamsters as described previously (4). Mock control animals received DMEM alone. In humans, natural infectious doses are considerably lower: for instance, only 10 TCID50 units led to infection in 53% of participants in a human challenge study (5). For our experiments, the multiplicity of infection (MOI) can be calculated as MOI = Number of Infectious Units/ Number of Target Cells. Here, Infectious Units = $TCID_{50}$X 0.7 and estimated lung epithelial cells in hamsters $\approx 10^7$. Then MOI= $(10^5 \times 0.7)/10^7 = 0.007$, which is 100–1000 times higher than typical human exposures. This higher dose is standard in animal models and is used to achieve consistent infection (2, 4, 6).

Cardiac injection of PIC in guinea pigs followed a method we described previously (7). Briefly, 450-500g male Hartley guinea pigs (Hilltop Lab Animals, Inc.) were anesthetized with 4% isoflurane for 4 min, intubated, and ventilated with oxygen and 2% isoflurane for direct myocardial injection. 200 µl PBS with 100 µg PIC was intramuscularly injected into the ventricular wall at multiple sites. Vehicle-injected controls received only PBS injection. For isolated adult cardiomyocyte Ca^{2+} transient studies (Fig. 4H), we used an adenoviral-GFP vector to mark isolated cells near the injection site to control for possible effects of the injection itself, apart from those caused by PIC. A 100 µl solution containing 1×10^{10} PFU adenoviral vector expressing GFP was injected into the ventricular wall around the apex in the absence (Control: AdV-GFP) or presence of 50 µg PIC (PIC+AdV-GFP). Cardiomyocytes were isolated as described previously (7), at 3 days post-injection.

Radiotelemetry device and osmotic pump implantation

Device implantation performed 4 days before the SARS-CoV-2 infection. 6- to 8-week-old male hamsters were anesthetized with 4% isoflurane and maintained with oxygen and 2% isoflurane during surgery. To measure subpleural pressure, the esophagus was isolated approximately 1.5 cm from its junction with the diaphragm and a 25-gauge needle was inserted between the serosal and muscularis layers. The needle was tunneled past the junction with the diaphragm into the thoracic cavity. The needle was then removed and the transmitter catheter of the dual pressure/biopotential device (model HD-X11, DSI) was threaded through the tunnel and secured in place with suture on the serosal layer. To monitor the ECG, the biopotential leads (HD-X11 or ETA-F10) were secured with suture on the muscle layer at Lead II position.

The body of the device was placed on the side of abdominal cavity. For treatment with mitoTEMPO or Ruxo, Alzet osmotic pumps filled with drug or vehicle were implanted into abdominal cavity to deliver the drug for 14 days. Both mitoTEMPO and Ruxo were delivered at 1 mg/kg/day. After 3 days of recovery, baseline ECG and subpleural pressure were recorded for 24 hr. Then the hamsters were sedated intramuscularly with xylazine and ketamine and inoculated intranasally with SARS-CoV-2. Mock-infected animals were inoculated with DMEM alone. ECGs were recorded for 24 hr at 1, 3, 5, 7, 14, 21, and 28 dpi. Hamsters were euthanized at 4 or 28 dpi. Body weights were measured daily until 10 dpi, then on 14, 21, and 28 dpi.

Implantation of the biopotential devices (Model ETA-F10, DSI) in guinea pigs was performed in the same surgical session as the PIC injection. ECGs were recorded at 2-3 days after injection and the animals were then euthanized for heart tissue collection.

ECG and subpleural pressure data were collected with Ponemah 3.0 (DSI, Inc.). AV block was manually counted for a 2-hour duration from 0-2am. Mean RR interval and frequency of RR interval longer than meanRR+100ms for hamster, or meanRR+2xS.D. for guinea pig, were analyzed with custom-written Matlab routines (Mathworks, Inc.), available at https://github.com/borourkelab/OpenPonemah. Subpleural pressure and HRV were analyzed with LabChart 8 (AD Instruments, Inc.) with Modules of Pressure and HRV.

Cultured cell models

hiPSC-CM were differentiated according to previously established protocols (8). The hiPSC-CM were then enriched to >95% purity using Miltenyi's MACS purification protocol. Briefly, hiPSC-CM on day 10-14 of differentiation were trypsinized and collected in a tube. Dead cells were removed using a dead cell removal kit (Catalog No. 130-090-101, Miltenyi Biotec). Cardiomyocytes were purified using a PSC-Derived Cardiomyocyte Isolation Kit (130-110-188, Miltenyi Biotec). LS columns were used to enrich cardiomyocytes (130-042-401, Miltenyi Biotec). Purified cardiomyocytes were plated in monolayers onto Geltrex™ coated (Cat. No. A1413202, ThermoFisher Scientific) plates for further experiments. A549 lung carcinoma epithelial cells were used to study effects of innate immune activation in a human lung-derived cell model. For hiPSC-derived nodal cells with atrial cells, differentiation of WTC11 hiPSCs was initiated by changing the stem cell medium to RPMI + B27 (no insulin) with 6 µM CHIR99021 on day 0. On day 2, the medium was changed to RPMI + B27 (no insulin) with 2 µM IWR1. On day 3, all-trans retinoic acid (RA, 1 µM final) was added. On day 5, media was refreshed with RPMI + B27 (no insulin) containing 1µM RA. The following day, the medium was refreshed with RPMI + B27 (no insulin). Medium was refreshed with RPMI + B27 on day 8 and every other day thereafter. The sinoatrial nodal monolayers typically began beating on day 12.

Polyinosinic:polycytidylic acid activation of innate immune signaling

Polyinosinic:polycytidylic acid (PIC) is a dsRNA mimetic known to activate the intracellular pattern recognition receptors OAS/MDA5/RIG-I to trigger IRF3-mediated signaling, leading to the upregulation of Type I Interferons. PIC (Cat. No. P1530, Sigma) was reconstituted in saline at 10mg/ml or 20mg/ml and used at 200µg/ml in our cellular experiments. In A549 cells, PIC treatment alone elicited a weak interferon response (indexed by STAT1, pSTAT1, OAS1,2,3 protein expression) that was markedly accentuated by co-addition of an empty replication-incompetent adenovirus vector (AdV; Supplement Fig. S1A). This effect was likely due to enhanced AdV-induced endocytosis (9) and internalization of PIC, as it could be mimicked

by lipofectamine treatment. AdV alone did not activate the immune response and, unlike lipofectamine, was not cytotoxic, so subsequent immune challenges in A549 cells were performed with the combination of PIC+AdV. However, in hiPSC-CM, PIC alone was sufficient to activate an immune response, with a STAT1 and pSTAT1 increase (Supplement Fig. S1B), and addition of AdV did not enhance the response. Therefore, studies of hiPSC-CM were performed with PIC alone.

Protein extraction and Immunoblotting

Ventricles and lungs were harvested at 4 or 28 dpi. Tissues were rinsed in cold PBS, rapidly heat-stabilized (Stabilizor™, Denator, Inc.), snap-frozen in liquid nitrogen, and stored in a -80 freezer. To extract protein, stabilized tissues were homogenized with RIPA buffer in the presence of 2% SDS, solubilized, and boiled in 1x LDS sample buffer for SDS-PAGE. The protein mixture was separated on a 4-12% NuPAGE gel (1 mm, Invitrogen). Samples were run at room temperature for 35 min at 200 V. Proteins were transferred to nitrocellulose membranes with iBlot (Invitrogen, Inc.), using program 3 for 7 min. Membranes were stained with Ponceau S solution (Sigma-Aldrich) to evaluate the transfer efficiency. Membranes were blocked for 1hr using Odyssey® blocking buffer (Li-Cor Biosciences) and incubated with the primary antibody (Antibodies listed in Supplementary Table 1) overnight at 4°C. Antibody binding was visualized with an infrared imaging system using IRDye secondary antibodies (800CW Donkey anti-Rabbit IgG, #926-32211 and 680RD Donkey anti-Mouse IgG, #926-68072; Odyssey, Licor Biosciences) and quantification of band intensity was performed using the Odyssey Application Software 3.0.

hiPSC-CM, A549 cells, or EHTs were collected 96 hr after treatment with PIC (200μg/ml), PIC+AdV, PIC+Ruxo (1μM), or PIC+mitoTEMPO (1μM) along with an untreated control group. Four days after treatment, samples were washed in ice-cold PBS and lysed in RIPA buffer (Cat. No. R0278, Sigma), supplemented with protease (Cat. No. P8340 or Cat. No. 11836170001 Sigma) and phosphatase (Cat. No. P0044 or Cat. No. 4906845001, Sigma) inhibitor cocktail. EHTs were snap-frozen in liquid nitrogen and stored at -80°C before lysis. Lysates were sonicated and centrifuged, and the supernatants were collected to perform immunoblotting. Protein samples were denatured at 70° C for 10 min after combining with 5% β-mercaptoethanol and 1X NuPAGE™ LDS Sample Buffer (Cat. No. NP0007, ThermoFisher) and run in 4-12% Bis-Tris gels (Cat. No. WG1402BOX or NP0322BOX). Proteins were transferred to nitrocellulose membranes using iBlot transfer stacks (Cat. No. IB301001). Membranes were blocked with Intercept® TBS blocking buffer (Cat. No. 927-60001, LICOR) for 1 hour at room temperature. Primary antibodies (diluted 1:1000 in blocking buffer) were incubated overnight at 4°C and secondary antibodies (IR Dye 800w goat anti-rabbit, Cat. No. 926-32211, LI-COR) were incubated at 1:10000 dilution in Intercept® blocking buffer for 1hr at room temperature, and detected using the LICOR-Odyssey system. Band intensities were quantified using the Fiji implementation of NIH ImageJ (10). Normalization for protein loading was based on intensity staining of the membrane with Ponceau (see Supplemental Data). Relative band intensity reports fold-change with respect to control. Data were analyzed for normality using Shapiro-Wilk test. For normally distributed data, a parametric test was used. Those that did not pass the normality test were tested with non-parametric significance tests.

Cytokine Proteome Profiler

Cell-culture medium samples were collected 96 hr after PIC, PIC+Ruxo, or PIC+mitoTEMPO treatments. Cytokines released from hiPSC-CM were analyzed using the Proteome Profiler Human XL Cytokine Array Kit (Cat. No. ARY022B R&D Systems). Chemiluminescence from the membrane was detected and scanned

on an iBright Imaging System FL1000 (Thermo Fisher Scientific). Scanned images were analyzed using Quick Spots image analysis software, version 25.5.2.3 (Ideal Eyes Systems).

Multielectrode Array Analysis

Approximately 50,000 hiPSC-CM (ventricular) were plated onto each Geltrex-coated well of a 24-well Cytoview MEA plate (Axion Biosystems, cat# M384-tMEA-24W) and maintained in RPMI+B27(+insulin) media for 48 hours to stabilize. Baseline field potential measurements were recorded on a Maestro Edge™ system (Axion Biosystems). After baseline readings, the medium was replaced with RPMI+B27(+) containing PIC (200 µg/ml) or RPMI+B27(+) alone as the control. Electrophysiological parameters were recorded at 24, 48, 72, 96, and 120 hours post-treatment. 50,000 hiPSC-derived atrial and nodal cells were plated on a fibronectin-coated 96-well plate for the Multiwell-MEA-system (96W700/100F-288 Multichannel systems). Field potentials began on day 7 after plating (37°C; 5% CO_2). The cells were treated with PIC (200 µg/ml) or with RPMI+B27(+) alone as the control after measuring spontaneous electrical signals. Recordings were taken at 24, 48, 72 and 96 hours post-treatment. Signals were collected at 20 kHz, High-pass-filtered at 1 Hz, and low pass-filtered at 3.5 kHz and analyzed with Multiwell-Analyzer (Multi Channel Systems).

Ca^{2+} transient and Conduction Velocity measurements in hiPSC-CM

hiPSC-CM (250,000/well) were plated onto 8-well plastic plates (ibidi, Inc. Cat. No. 80806). hiPSC-CM were loaded with 2µM Fluo-4 AM (Catalog No. F14201, Invitrogen) at 37°C for 15 minutes. Cells were washed once with PBS before transfer to Tyrode's buffer (130 mM NaCl, 5 mM KCl, 1 mM $MgCl_2$, 10 mM NaHEPES, 1 mM $CaCl_2$ and 5 mM Glucose) for imaging. hiPSC-CM Ca^{2+} transients were imaged on a spinning disk-confocal microscope (Andor Revolution, Olympus IX-70). Images were collected at 20X magnification (Olympus UCPlanFL N objective) using 488nm laser excitation (35% power), at 33.3 frames/sec for 22-25 seconds. Custom-written ImageJ and Excel-based macros were used to analyze Ca^{2+} transient dynamics. To obtain conduction velocities of PIC-treated and control hiPSC-CM, myocytes were plated on 12 well plates and optical mapping was performed using the voltage-sensitive dye, FluoVolt™ (#F10488, ThermoFisher). Using a standard GFP filter set, electrical propagation was recorded as 10-second videos from spontaneously beating monolayers. Conduction velocities were calculated across a 20mm diameter area as described earlier (11).

Engineered Heart Tissues

Engineered heart tissues (EHTs) were fabricated using the Curi Bio Mantarray™ platform, as previously described (12). Briefly, a mixture of $1x10^6$ hiPSC-CM (Celo.Cardiomyocytes, Celogics, Inc.), $5x10^4$ HS-27A human bone marrow stromal cells (ATCC), and 0.5mg human fibrinogen (Sigma-Aldrich) in a total volume of 100µL EHT culture media (Celo.Cardiomyocyte advanced culture media, B27, 5g/L aminocaproic acid) with 10µM ROCKi was prepared per tissue. This mixture was pipetted into Mantarray casting wells containing 50µL EHT culture media with 0.3U human thrombin (Sigma-Aldrich). EHTs were then incubated for one hour at 37°C, at which point 1mL of EHT culture media with 10µM ROCKi was added to each casting well. After overnight incubation at 37°C, EHTs were transferred to 2mL/well EHT culture media. EHT culture media was changed every 2-3 days thereafter.

Sarcomere shortening and Ca^{2+} transient recordings of adult cardiomyocytes

Ventricular myocytes were loaded with 3 µM Fura2-AM (Invitrogen, Molecular Probes, Carlsbad CA) in a modified Tyrode's solution containing (in mM) NaCl 138, KCl 4, $CaCl_2$ 2, $MgCl_2$ 1, HEPES 10, NaH_2PO_4 0.33, and Glucose 10 (pH 7.4 with NaOH) for 15min. After rinsing, cells were placed in a perfusion chamber with a flow-through rate of 2 ml/min, and sarcomere length and whole cell Ca^{2+} transients were recorded using an inverted fluorescence microscope (Nikon, TE2000), and IonOptix (Myocam®) software.

Tissue immunofluorescence

Tissues were fixed with 4% paraformaldehyde for 3 days and transferred to 30% sucrose for 24-48 hours at 4°C, followed by OCT embedding. For immunofluorescence, frozen sections were washed with PBS, blocked with 2% BSA and 0.05% triton X-100 in PBS for 1hr, and incubated with primary antibodies at 4°C overnight and secondary antibodies for 1 hr. Images were acquired with a spinning-disk confocal microscope (Andor Revolution) and analyzed using Fiji (10). For quantitative analysis, slides were prepared under identical staining conditions and all images were collected under exactly the same imaging conditions (i.e., exposure, magnification, detector settings, etc.). To avoid any subjective input of the user, the unmodified raw images were analyzed using Fiji's automated thresholding function for particle analysis of the objects. Object densities were normalized to tissue area. For connexin 43 (Cx43) analysis in the in vivo models, we found that in the hamster heart under control conditions, Cx43 was present at the intercalated discs, intracellular locations, and along the lateral borders of cardiomyocytes. In the COVID-19 group, its distribution was more intracellular; therefore, we quantified the percentage of the total Cx43 present at the intercalated disc, rather than gap junction lateralization. In control guinea pig hearts, Cx43 was mainly localized at the intercalated discs with little at the lateral membranes. After innate immune activation with PIC, lateral labeling was more distinct; therefore, we quantified Cx43 as the percentage of Cx43 signal exhibiting lateralized distribution.

qPCR

Total RNA was isolated from heat-stabilized hamster or guinea pig tissues using Trizol reagent, following manufacture's protocols. Equal amounts of RNA were transcribed into cDNA by High-Capacity cDNA Reverse Transcription Kit (Applied Biosystems). cDNA product was diluted 1:40 and real-time PCR was performed using FastStart SYBR Green Master Mix (Roche) on QuantStudio 5 (ThermoFisher), with primers specific to hamster genes encoding CCL2, CXCL10, CXCL11, IFNα, IFNβ, IFNγ, IL1b, IL6, IL10, OAS1, OAS2, OAS3, RIG1, TGFβ, TNFα as well as β-actin as an internal reference (primer sequences in Supplemental Table 2). For hiPSC-CM, RNA was extracted with RNAmini Plus Qiagen kit. qPCR was performed with TaqMan™ RNA-to-C_T™ *1-Step* Kit (Applied Biosystems, #4392938) and Taqman probes for Connexin 43 (#Hs00748445) and Connexin 45 (Hs00271416), with B2M (Hs00187842) as the internal reference gene.

Statistical analysis

Values are expressed as mean±SEM. Statistical analyses between 2 groups or among multiple groups were performed with Student's t-test or 1-way ANOVA using GraphPad Prism version 10.0.2 for MacOSX. The time series data from the hamster studies were analyzed with parametric (Fig. 1, 2B, 8A-D) or non-parametric methods (Fig. 2C-E, 8E-G), depending on the normality of the data distribution. Parametric analysis was performed using JMP Pro 16.0. The responses of body temperature, respiration, and RR intervals to SARS-CoV2 infection, with or without treatment, within each group were analyzed with 1-way ANOVA with repeated measures followed by Dunnett's test to compare each time point with baseline

(Day 0). The comparisons among groups were analyzed with multiple regression followed by Tukey HSD test for pairwise comparison. Non-parametric analysis was performed using R. The time course response within each group was analyzed with Friedman's test and the comparison among groups was analyzed with the Kruskal-Wallis test. Conover test was used for pairwise comparison in both methods. ECG signals, recorded for 24 hours for each time point, were analyzed by 6-hour segment. RR interval was analyzed with a nested model of multiple regression. In non-parametric analyses, the parameters of the 6-hr segments were averaged for each time point. Sample size is provided in the figure legends. An extended statistical analysis for cross comparisons between for different treatments and time points is presented in the supplement (Supporting Data Values File).

Literature Cited:

1. Frere JJ, et al. SARS-CoV-2 infection in hamsters and humans results in lasting and unique systemic perturbations after recovery. *Sci Transl Med*. 2022;14(664):eabq3059.

2. Imai M, et al. Syrian hamsters as a small animal model for SARS-CoV-2 infection and countermeasure development. *Proceedings of the National Academy of Sciences*. 2020;117(28):16587–16595.

3. Yuan S, et al. Pathogenicity, transmissibility, and fitness of SARS-CoV-2 Omicron in Syrian hamsters. *Science*. 2022;377(6604):428–433.

4. Mulka KR, et al. Progression and Resolution of Severe Acute Respiratory Syndrome Coronavirus 2 (SARS-CoV-2) Infection in Golden Syrian Hamsters. *Am J Pathol*. 2022;192(2):195–207.

5. Killingley B, et al. Safety, tolerability and viral kinetics during SARS-CoV-2 human challenge in young adults. *Nat Med*. 2022;28(5):1031–1041.

6. Chan JF-W, et al. Simulation of the Clinical and Pathological Manifestations of Coronavirus Disease 2019 (COVID-19) in a Golden Syrian Hamster Model: Implications for Disease Pathogenesis and Transmissibility. *Clinical Infectious Diseases*. 2020;71(9):2428–2446.

7. Liu T, et al. MCU Overexpression Rescues Inotropy and Reverses Heart Failure by Reducing SR Ca2+ Leak. *Circulation Research*. 2021;128(8):1191–1204.

8. Hawthorne RN, et al. Altered Electrical, Biomolecular, and Immunologic Phenotypes in a Novel Patient-Derived Stem Cell Model of Desmoglein-2 Mutant ARVC. *JCM*. 2021;10(14):3061.

9. Nestić D, et al. The Revolving Door of Adenovirus Cell Entry: Not All Pathways Are Equal. *Pharmaceutics*. 2021;13(10):1585.

10. Schindelin J, et al. Fiji: an open-source platform for biological-image analysis. *Nat Methods*. 2012;9(7):676–682.

11. Wolfson DW, et al. Transient pacing in pigs with complete heart block via myocardial injection of mRNA coding for the T-box transcription factor 18. *Nat Biomed Eng*. 2024;8(9):1124–1141.

12. Tsui JH, et al. Tunable Electroconductive Decellularized Extracellular Matrix Hydrogels for Engineering Human Cardiac Microphysiological Systems. *Biomaterials*. 2021;272:120764.

Supplement Table 1- List of Antibody Sources

Antibody	usage	host	Catalog #	Vemdor	dilution
IBA1	IF	rabbit	A1527	Abclone	1:200
Contactin2	IF	Goat	AF-4439	R&D	1;50
CD163	IF	Rabbit	AB182422	Abcam	1:100
SARS-CoV2 nucleoCapsid	IF/W	rabbit	40143-R001	Sino Biol.	1:500/1:2000
Anti-sheep, FITC	IF	Donkey	STAR88D488	BioRad	1:400
anti-rabbit, TRITC	IF	Goat	A16123	ThermoFisher	1:800
anti-Rabbit, 800CW	W	Goat	926-32211	Li-Cor	1:20000
Phospho-IRF-7	W	rabbit	12390S	Cell Signaling Technology	1:1000
IRF-7	W	rabbit	13014S	Cell Signaling Technology	1:1000
Phospho-STAT1	W	rabbit	8826	Cell Signaling Technology	1:1000
STAT1	W	rabbit	14994	Cell Signaling Technology	1:1000
IRF-9	W	rabbit	76684	Cell Signaling Technology	1:1000
MX1	W	rabbit	37849	Cell Signaling Technology	1:1000
OAS1	W	rabbit	14498	Cell Signaling Technology	1:1000
OAS2	W	rabbit	54155S	Cell Signaling Technology	1:1000
OAS3	W	rabbit	41440S	Cell Signaling Technology	1:1000
Cx43	IF	rabbit	3512S	Cell Signaling Technology	1:100

Supplement Table 2- Hamster primers

gene	Forward Primer	reverse primer
b-actin	CCA GAG CAA GAG AGG TAT T	TCG TTG TAG AAG GTG TGG
CCL2	GAA AGA TCC CAG AGA AGA G	CTT GAG CTT GGT GAT GA
CXCL10	GTG ACC TGT GGA TTG TTG	TTC TGG CTC TTC CTG TAT AA
CXCL11	CGC CTC ATA CGG GAA ATG TAT	CAT CAG ACA CTC CCT GGT TTC
IFNa	TCC CAC CAA CTC ACT ATA C	CAA GAG GAT TCC GTG ATA TTT
IFNb	TAT CCC TGT CCA TCA ACT AC	CAC CTC CAT AGG CAT CTT
IFNy	TGT TGC TCT GCC TCA CTC AGG	AAG ACG AGG TCC CCT CCA TTC
IL-1b	CTG AAA GCT CTC CAT CTC	GCC ACA GGT ATC TTG TT
IL-6	GGA CAA TGA CTA TGT GTT GTT AGA A	AGG CAA ATT TCC CAA TTG TAT CCA G

IL-10	GGT TGC CAA ACC TTA TCA GAA ATG	TTC ACC TGT TCC ACA GCC TTG
OAS1	TCT CCA AGG TGA TGA AGG	GCT GGT GAG ATT GTT AAG G
OAS2	CAC CAT GAG AAG TAC AAT AAG	ATC AAA GGC TGG AAG TAG
OAS3	GCT GCC CTC TAG TTA TG	GTC CTT GTT CTG TTG GA
RIG1	GTG ACC TGT GGA TTG TTG	TTC TGG CTC TTC CTG TAT AA
TGFb	GGC TAC CAC GCC AAC TTC TG	GAG GGC AAG GAC CTT ACT GTA CTG
TNFa	TGA GCC ATC GTG CCA ATG	AGC CCG TCT GCT GGT ATC AC
Sars-CoV2 delta Spike	CCA CAA AAA CAA CAA AAG TTG G	TGA GAG ACA TAT TCA AAA GTG CAA

Supplement Table 3- hiPSC-CM-Cytokine Array

	PIC(n=5)	SEM	+Ruxo(n=2)	SEM
Adiponectin/Acrp30	1.2085628	0.067297	1.084691	0.043779
Angiogenin	2.1054212	0.966669	0.91118	0.094981
Angiopoietin-1	2.2785056	0.697605	0.925771	0.064425
Angiopoietin-2	1.332314	0.106618	1.05127	0.140703
Apolipoprotein A-1	1.4524768	0.074532	0.875808	0.103923
BAFF/BLyS/TNFSF13B	2.3284252	0.743817	1.080638	0.137438
BDNF	1.5601756	0.16595	1.047205	0.132103
C-Reactive Protein/CRP	1.3488904	0.082289	0.912717	0.070433
CCL17/TARC	1.4424288	0.061948	0.965699	0.222105
CCL19/MIP-3 beta	3.0288266	1.251598	1.113481	0.259478
CCL2/MCP-1	3.1723178	0.915611	0.83498	0.00285
CCL20/MIP-3 alpha	15.5656698	13.0605	1.221683	0.30985
CCL3/CCL4 MIP-1 alpha/beta	19.7142228	15.0717	1.440288	0.263417
CCL5/RANTES	17.3851902	5.397992	1.390715	0.193217
CCL7/MCP-3	4.6860314	3.252532	0.838003	0.068895
CD14	1.6636062	0.232924	1.109644	0.27837
CD30	1.333497	0.169473	1.064739	0.049854
CD31	1.3960622	0.161299	1.338063	0.485993
CD40 ligand	1.2925922	0.121816	1.081436	0.077479
Chitinase 3-like 1	4.1547926	2.465684	0.702872	0.050424
Complement Component C5/C5a	1.6071846	0.23033	1.008338	0.154563
Complement Factor D	1.273009	0.065638	0.899029	0.056481
Cripto-1	1.6708122	0.298568	1.015132	0.080083
CXCL1/GRO alpha	6.3920874	1.860376	0.978544	0.035433
CXCL10/IP-10	89.180002	32.68489	1.218749	0.275235
CXCL11/I-TAC	41.7030432	33.17172	1.244408	0.385624

CXCL12/SDF-1 alpha	1.7287528	0.181067	1.040522	0.179947
CXCL4/PF4	1.605583	0.178836	1.260547	0.250934
CXCL5/ENA-78	13.9085766	9.885841	0.944036	0.023785
CXCL9/MIG	2.6364888	0.942528	1.396962	0.168094
Cystatin C	2.3061618	0.506042	1.151898	0.134713
Dkk-1	4.965894	1.474081	1.089239	0.057725
DPPIV/CD26	1.7739458	0.320999	1.021454	0.076379
EGF	2.1586222	0.713946	1.01189	0.062607
EMMPRIN	2.5718048	0.789068	1.123027	0.108206
Endoglin/CD105	1.5065322	0.203504	0.762404	0.024847
Fas Ligand	1.3421876	0.165382	0.90571	0.049545
FGF basic	1.4966196	0.105137	0.937225	0.065459
FGF-19	1.657135	0.210628	1.190624	0.115651
Flt-3 Ligand	1.8936294	0.353278	1.140806	0.040755
G-CSF	9.8155484	8.30316	1.128102	0.041315
GDF-15	4.8741136	1.763071	0.948506	0.068209
GM-CSF	1.6934694	0.378477	1.126109	0.061572
Growth Hormone (GH)	1.7356002	0.274018	0.810799	0.185592
HGF	4.2018714	2.007048	0.881435	0.128722
ICAM-1/CD54	2.1747818	0.557003	0.950961	0.0408
IFN-gamma	1.2768538	0.054068	0.970812	0.09473
IGFBP-2	1.377401	0.14277	1.040726	0.026047
IGFBP-3	4.034496	2.15735	1.102966	0.019377
IL-1 alpha/IL-1F1	1.2858292	0.088766	1.228784	0.106472
IL-1 beta/IL-1F2	1.7747486	0.362789	1.131139	0.035275
IL-10	1.5192256	0.099035	0.969178	0.086008
IL-11	1.4471918	0.095497	1.009244	0.100844
IL-12 p70	1.3750594	0.10219	1.093594	0.085481
IL-13	1.696196	0.339707	1.206015	0.103586
IL-15	1.9582918	0.460879	1.08062	0.079593
IL-16	2.5615664	0.635304	1.087178	0.140808
IL-17A	1.9654252	0.418088	1.126834	0.163742
IL-18 BPa	15.7316144	13.57683	1.305137	0.355774
IL-19	1.4159508	0.169109	0.980446	0.016779
IL-1ra/IL-1F3	1.9606684	0.439469	1.048213	0.092304
IL-2	1.5766846	0.393835	1.141399	0.079841
IL-22	1.4119306	0.125007	0.782652	0.15752
IL-23	1.4587832	0.193636	0.934651	0.101298
IL-24	1.5186352	0.104092	0.97378	0.077665
IL-27	1.4768848	0.117661	1.159607	0.262546
IL-3	1.4575902	0.34632	1.139685	0.147909

IL-31	1.5169752	0.212865	1.060714	0.190246
IL-32 alpha/beta/gamma	1.4680872	0.154683	1.152776	0.121204
IL-33	1.665933	0.329233	1.233423	0.003236
IL-34	2.3314664	0.545274	1.235241	0.091049
IL-4	1.5043808	0.171366	0.997863	0.026452
IL-5	1.2458654	0.137511	0.799691	0.180663
IL-6	10.3800752	7.209792	0.93069	0.099206
IL-8	11.86945	4.245107	0.917451	0.036495
Kallikrein 3/PSA	1.679024	0.289404	1.422937	0.528298
KGF/FGF-7	1.6478468	0.209421	0.997695	0.044396
Leptin	1.4187688	0.148598	0.960115	0.013242
LIF	1.2874882	0.152914	0.806076	0.199003
Lipocalin-2/NGAL	1.396939	0.110544	0.929148	0.108902
M-CSF	1.5989998	0.212569	0.996407	0.158766
MIF	1.8563596	0.610488	1.205771	0.036568
MMP-9	6.5277048	2.745464	1.041292	0.289401
Myeloperoxidase	1.2290274	0.142744	0.938321	0.153145
Osteopontin (OPN)	2.9220832	1.044759	0.849462	0.228904
PDGF-AA	1.6000452	0.445003	0.899309	0.132587
PDGF-AB/BB	1.500799	0.074826	0.997536	0.155006
Pentraxin-3	2.5716456	1.054416	0.839467	0.027348
RAGE	1.8837892	0.363125	2.145745	1.015983
RBP4	2.0805912	0.439306	1.240603	0.149995
Relaxin-2	1.7922062	0.212301	1.415175	0.501026
Resistin	1.6785924	0.196995	1.021107	0.212882
Serpin E1/PAI-1	2.5615458	0.902751	0.797816	0.073465
SHBG	1.4661384	0.102449	1.112722	0.002808
ST2/IL-1 R4	1.2581856	0.073922	0.930787	0.23042
TFF3	1.4036804	0.058148	1.069102	0.279255
TfR	1.570087	0.278103	1.417126	0.55259
TGF-alpha	1.6747386	0.322899	1.630656	0.478111
Thrombospondin-1	1.4619158	0.26512	1.184769	0.051603
TIM-3	1.4701726	0.270349	1.65791	0.882127
TNF-alpha	1.5956582	0.279569	1.212706	0.101393
uPAR	2.348538	0.402858	1.365572	0.466394
VCAM-1	1.7173468	0.440618	1.103584	0.246384
VEGF	7.2772	2.599381	1.358042	0.576299
Vitamin D BP	1.211376	0.113223	1.188577	0.223708

Supplementary Figures

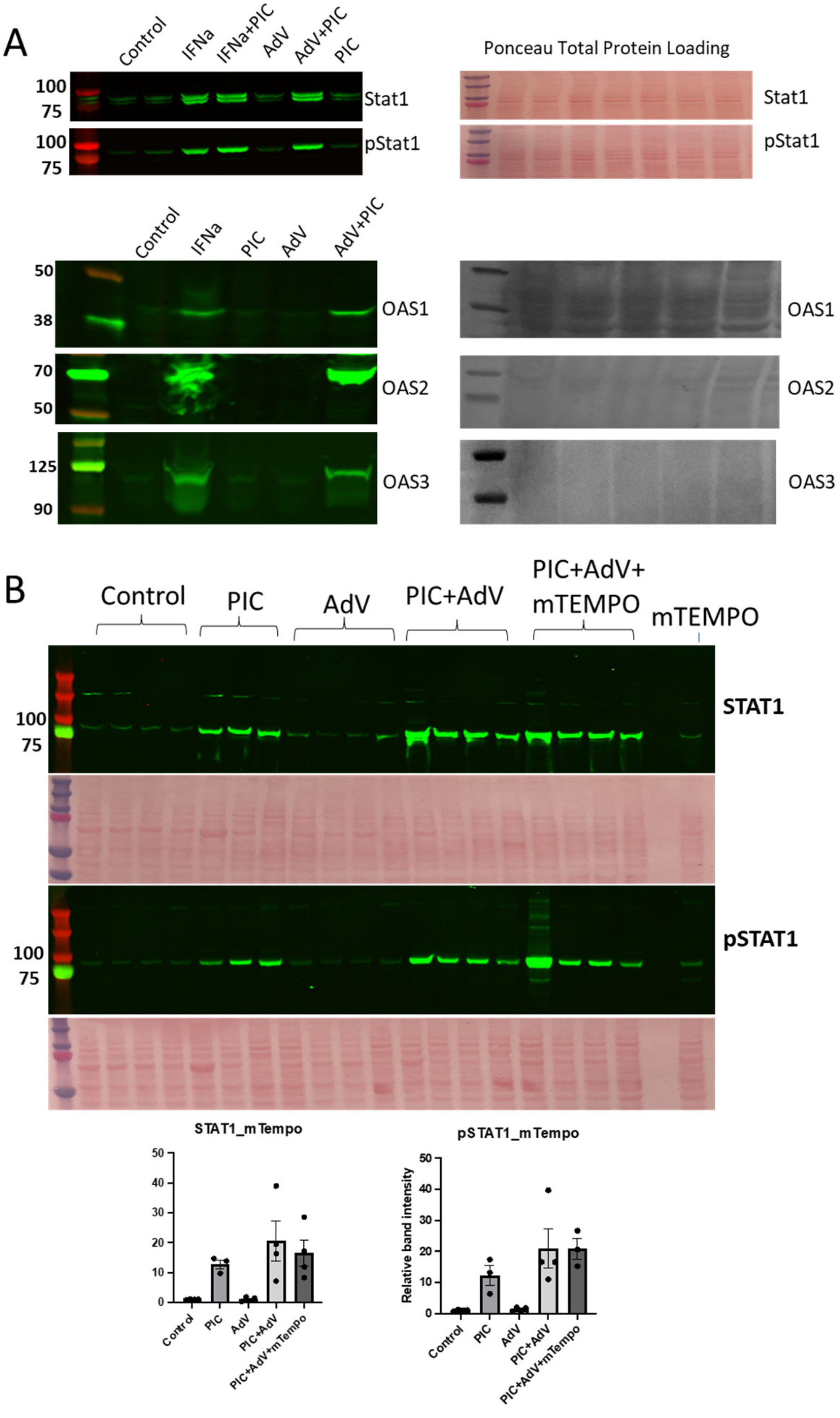

Supplementary Figure S1. A) In A549 lung epithelial cells, activation of interferon-stimulated protein expression by polyinosinic: polycytidylic acid (PIC) required co-application of an empty (no expression cassette) adenoviral vector (AdV). The combination PIC plus AdV increased STAT1 expression and phosphorylation (pSTAT), and expression of oligoadenylate synthase isoforms (OAS 1,2,3), but PIC or AdV alone did not. The response was also directly induced by the Type I interferon-alpha (IFNa), independent of the AdV. Ponceau-stained loading control gels are shown in the righthand panels. **B)** Human iPSC-derived cardiomyocyte innate immune responses could be induced by PIC alone and were not significantly enhanced by co-application of the AdV vector. Increased pSTAT1/STAT1 expression was not inhibited by mitoTEMPO treatment. Ponceau-stained loading controls appear below the western blots.

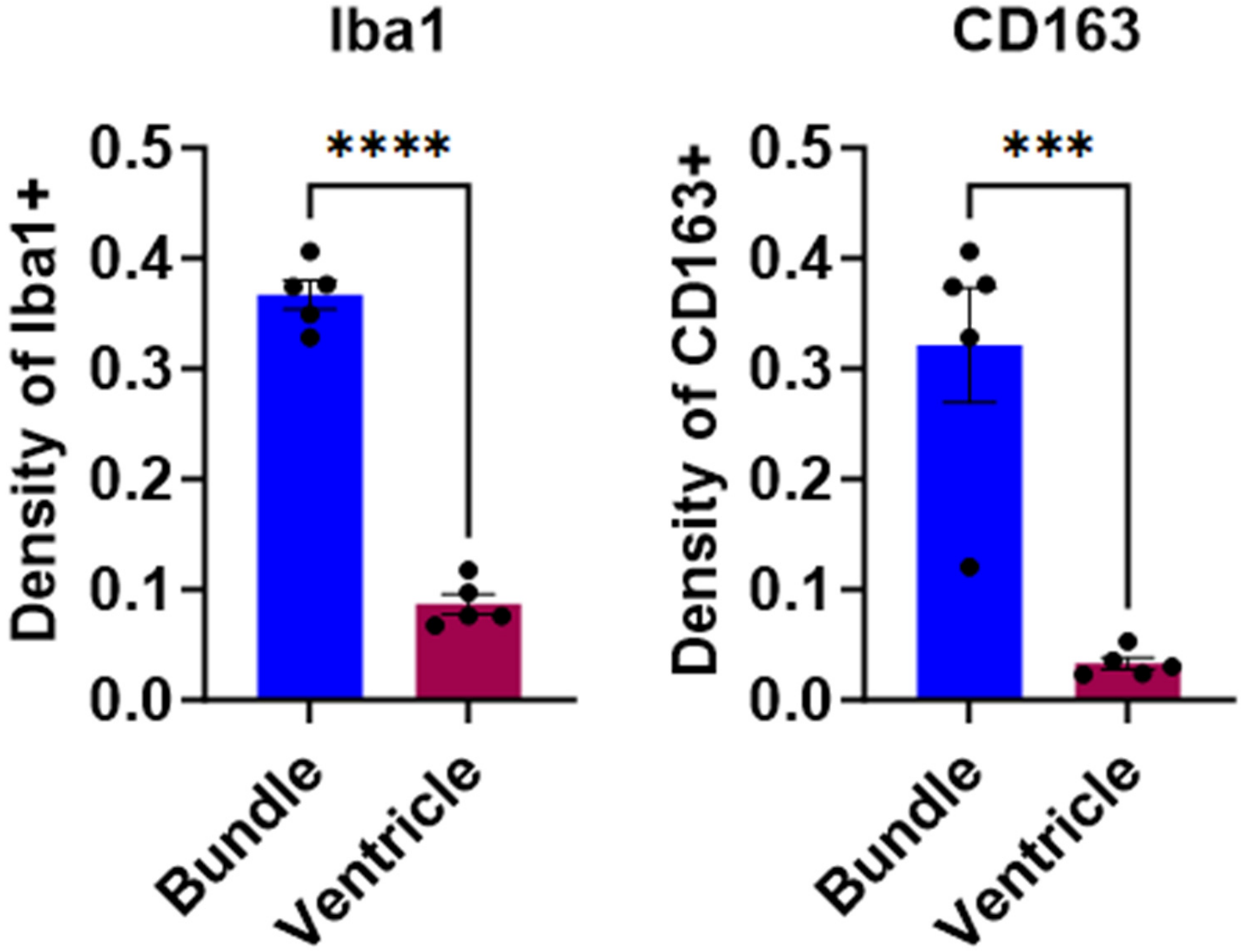

Supplementary Figure S2. Relative densities of Iba1+ and CD163+ macrophages were higher in the AV/bundle region compared to ventricles (Iba-1+ macrophages: 0.37±0.01 in bundle vs 0.09±0.01 in ventricles, $p<0.0001$; CD163+ macrophages: 0.16±0.02 in bundle vs 0.03±0.01 in ventricles, $p<0.005$ in mock-infected control hearts.

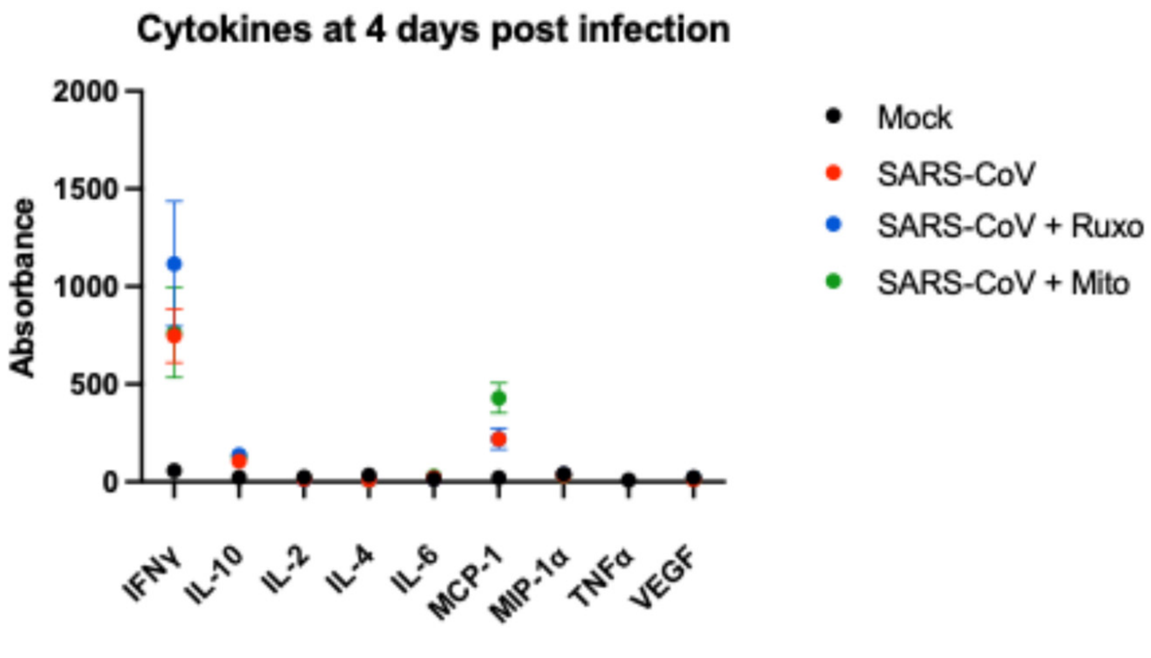

Supplementary Figure S3. Plasma cytokine levels 4 days post infection assessed by ELISA are shown. IFNγ and MCP-1 (CCL2) were significantly increased after SARS-CoV-2 infection.

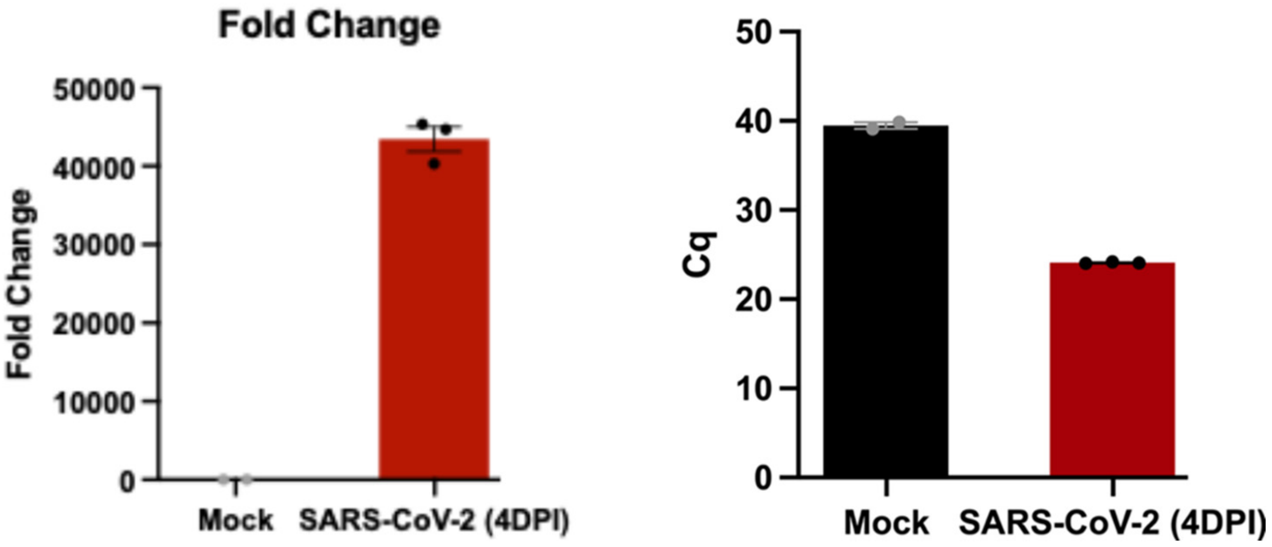

Supplementary Figure S4: SARS-CoV-2 viral RNA detected in serum of infected animals at increased levels. Serum from four mock-infected and four SARS-CoV-2-infected hamsters was pooled. RNA was extracted from 560uL of serum of mock and infected samples. Each dot represents technical replicates.

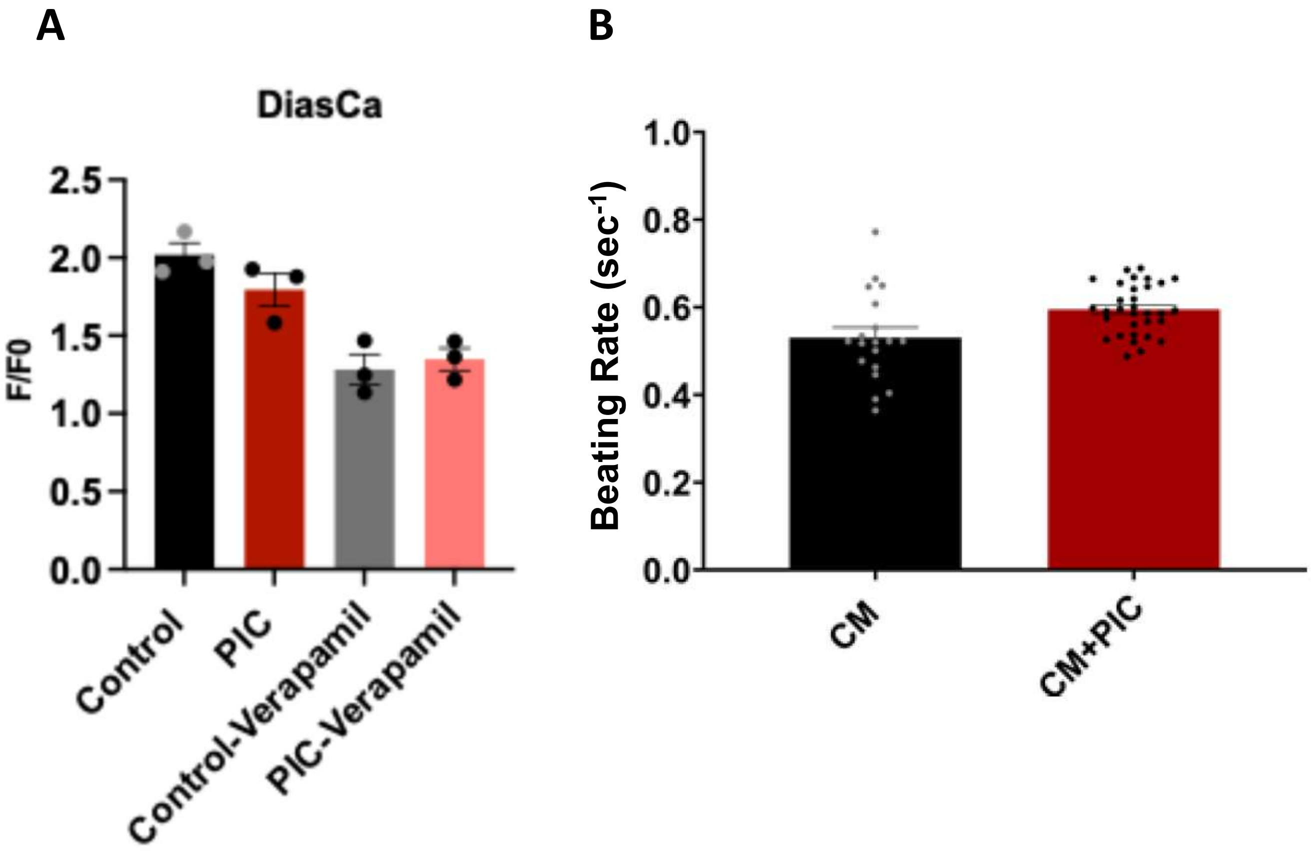

Supplementary Figure S5. Diastolic Ca^{2+} estimation and spontaneous beating rate of hiPSC-CM: A) We performed additional experiments with the calcium channel blocker verapamil, with the rationale that if we prolonged diastole at slower beating rates, we would allow the Ca^{2+} level to return to the lowest stable diastolic level possible. We then normalized our Fluo-4 signal to the lowest signal of verapamil-treated cells. There was no significant difference in diastolic Ca^{2+} in PIC-treated cells compare to controls, which indicates that the differences in Ca^{2+} transient amplitude observed were unlikely to be due to altered beating frequency affecting the diastolic Ca^{2+} Fluo-4 signal. **B)** Spontaneous beating rate was not significantly different from controls after PIC treatment in hiPSC-derived ventricular myocytes

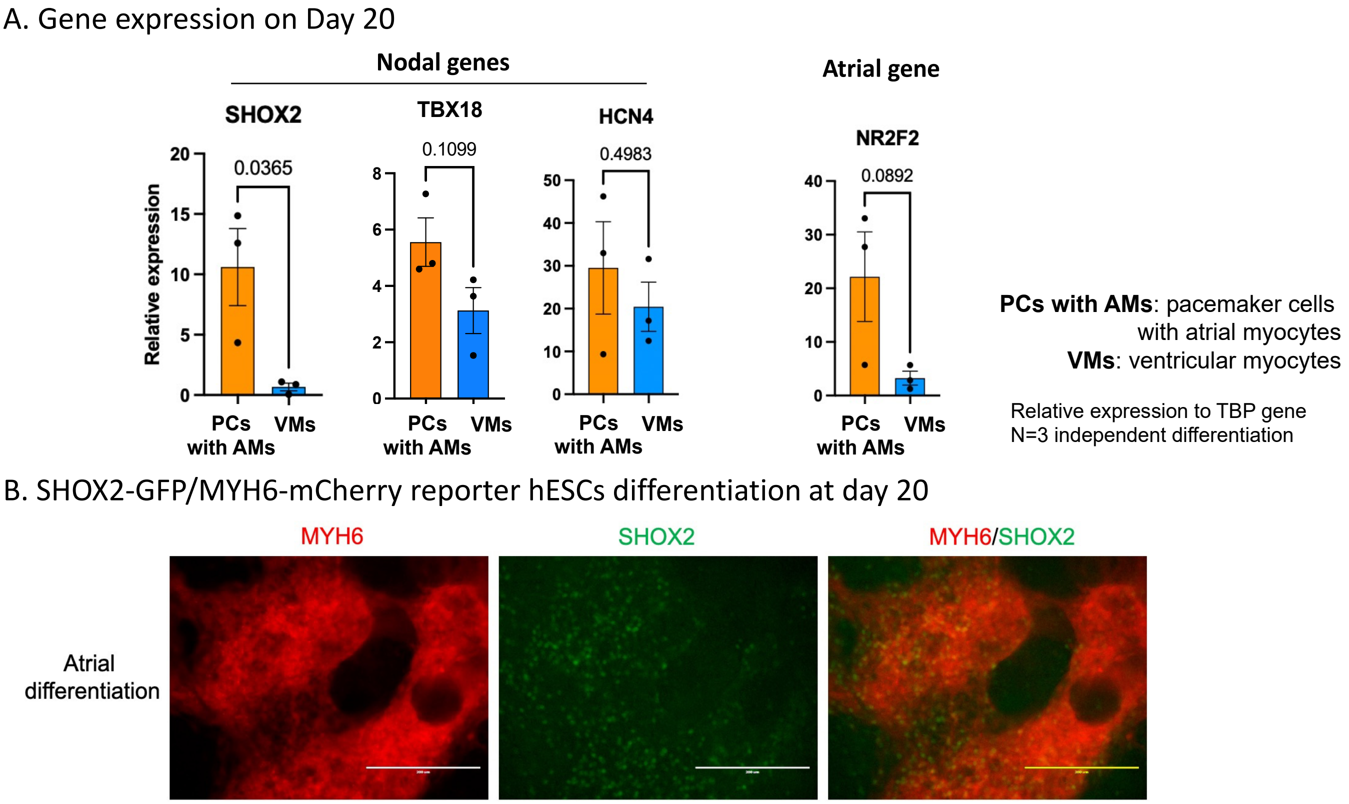

Supplementary Figure S6. A) hiPSC-derived sinoatrial nodal and atrial cell pacemaker/atrial monolayers (PM/AMs) showed increased expression of the nodal genes SHOX2 and TBX18, as well as increased expression of the atrial marker NR2F2, as compared to hiPSC-derived ventricular myocytes (VMs) **B)** Shox2 expression in pacemaker/atrial myocyte monolayers.

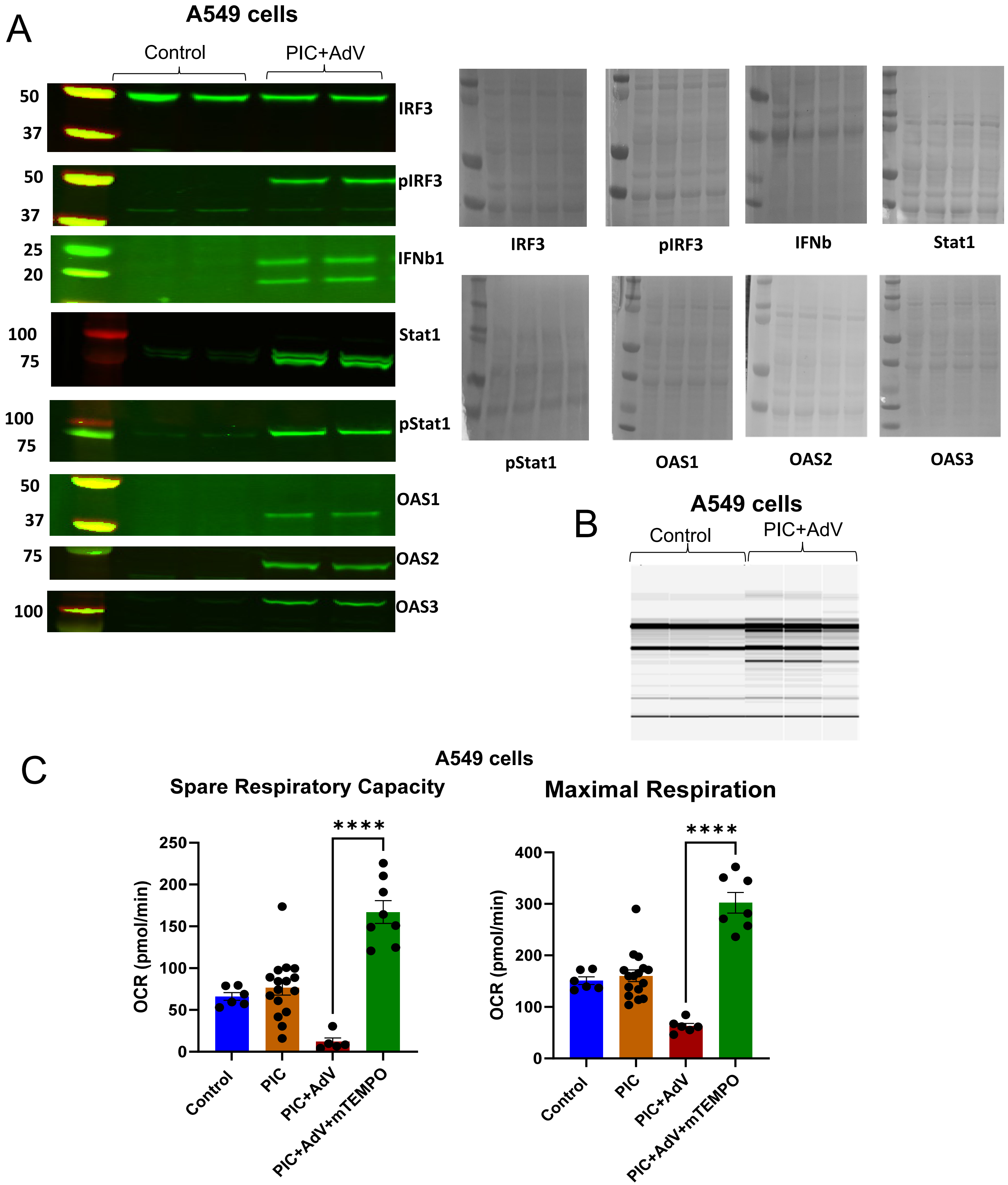

Supplementary Figure S7. PIC increases expression of Type-I interferon signaling pathway proteins, activates RNA degradation, and alters mitochondrial function in A549 cells. A) Activation of Type I interferon signaling pathway proteins (Panel A, left) and total protein loading by ponceau staining for western blots shown in Panel A, right. **B)** RNAse activation **C)** Mitochondrial Oxygen Consumption Rate (OCR; Seahorse XF96 assay) was suppressed after PIC+AdV treatment in A549 cells, including a decrease in both Maximal Uncoupled Respiration and Spare Respiratory Capacity. The mitochondrial deficiency was prevented by mitoTEMPO treatment, implicating mitochondrial ROS.

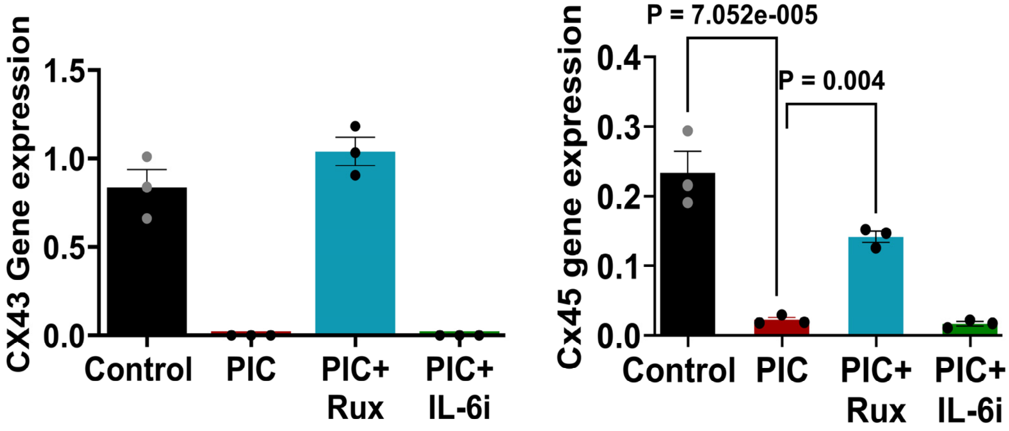

Supplementary Figure S8: Connexin 43 (Cx43) and 45 (Cx45) gene expression was suppressed by PIC and prevented by ruxolitinib treatment, but not by the IL-6 inhibitor, Tocilizumab (2.5 µg/ml) in hiPSC-CM.

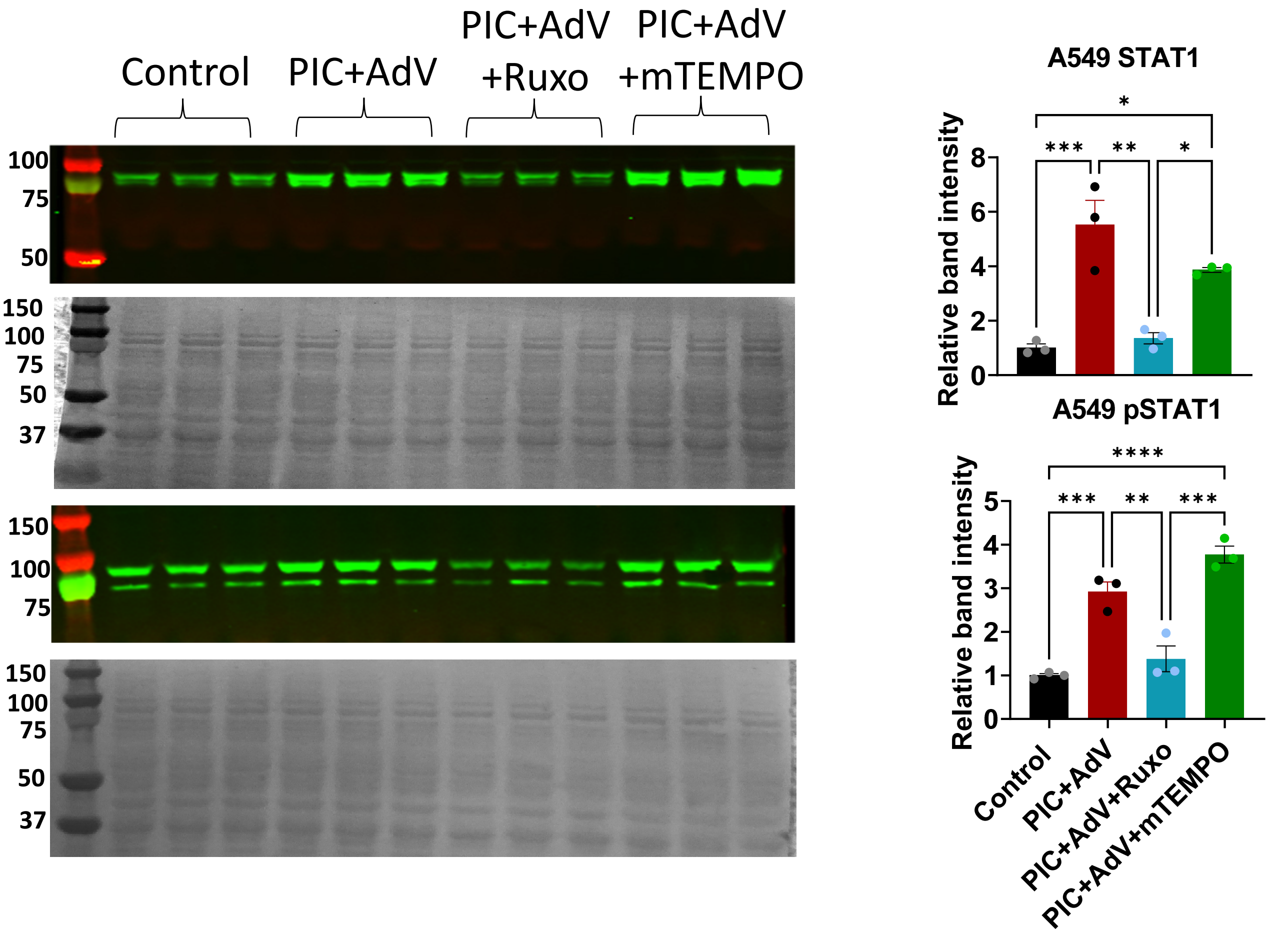

Supplementary Figure S9. In A549 cells, 2uM Ruxolitinib significantly suppressed PIC induced STAT1 and pSTAT1 response but mitoTEMPO did not. Relative band intensity normalized to Ponceau total protein loading for A549 cells (One-way ANOVA with $p<0.05$; **<0.005, ***0.0005, ****<0.0001)
