# Supplementary material for: Innate immune activation and mitochondrial ROS induce acute and persistent cardiac conduction system dysfunction after COVID-19
Source: JCI Insight. 2025 Dec 22;10(24):e193164. doi: 10.1172/jci.insight.193164 (PMC12890510; doi:10.1172/jci.insight.193164)
Supplement: Unedited blot and gel images [file jciinsight-10-193164-s036.pdf]

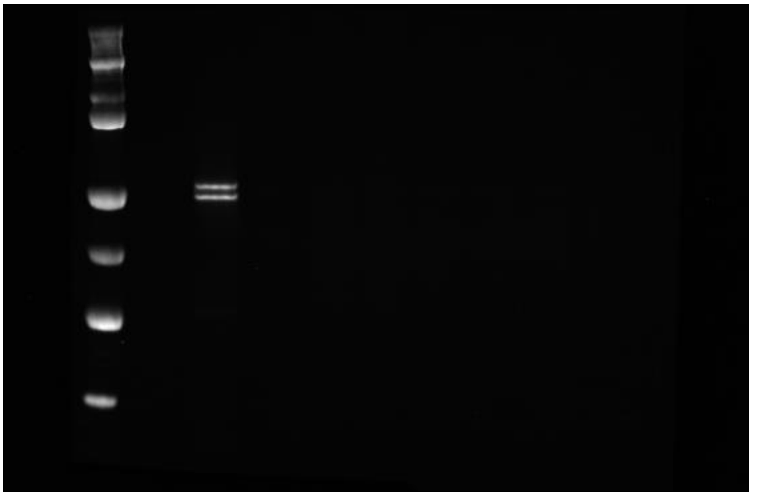

Full unedited gel for Fig. 3G
SARS-CoV-2
Ladder and subsequent 9 lanes included in Fig 3G

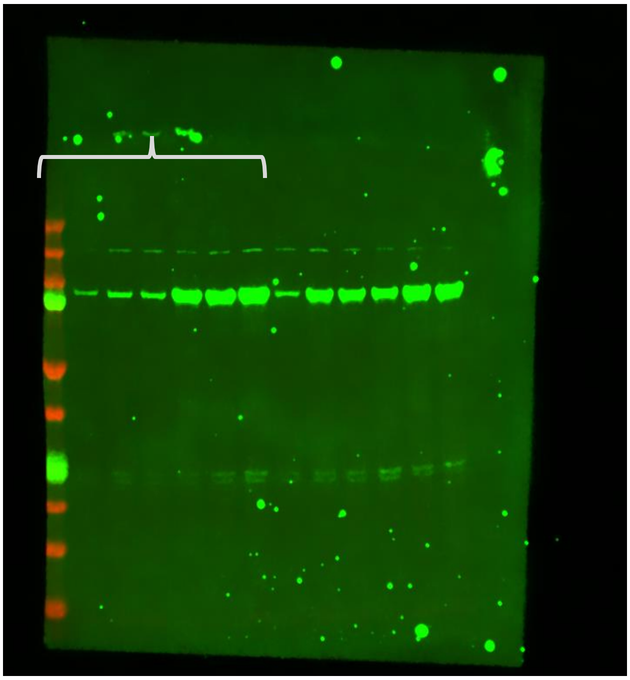

Full unedited gel for
Fig. 5A
STAT1
Ladder and
subsequent 6 lanes
included in Fig 5A

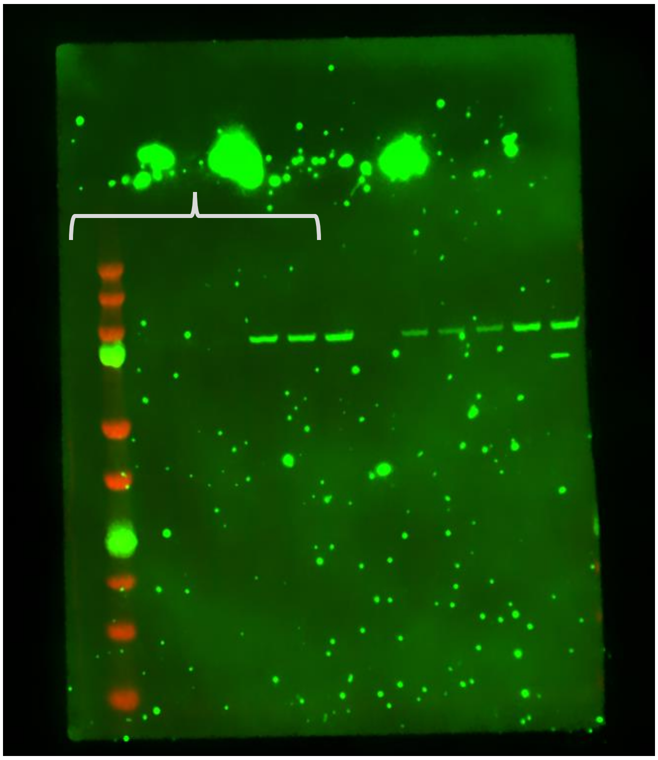

Full unedited gel for Fig. 5A
pSTAT1
Ladder and subsequent 6
lanes included in Fig 5A

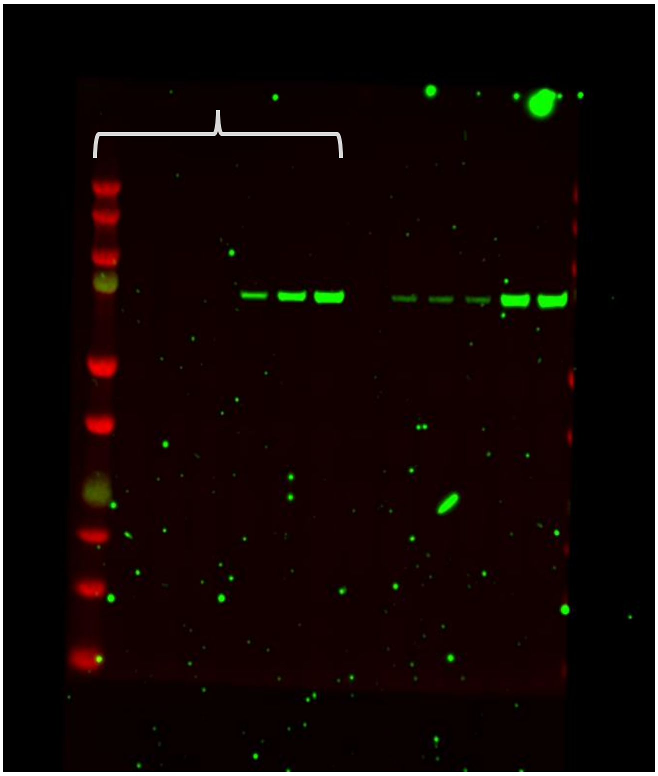

Full unedited gel for
Fig. 5A
MX1
Ladder and subsequent
6 lanes included in Fig
5A

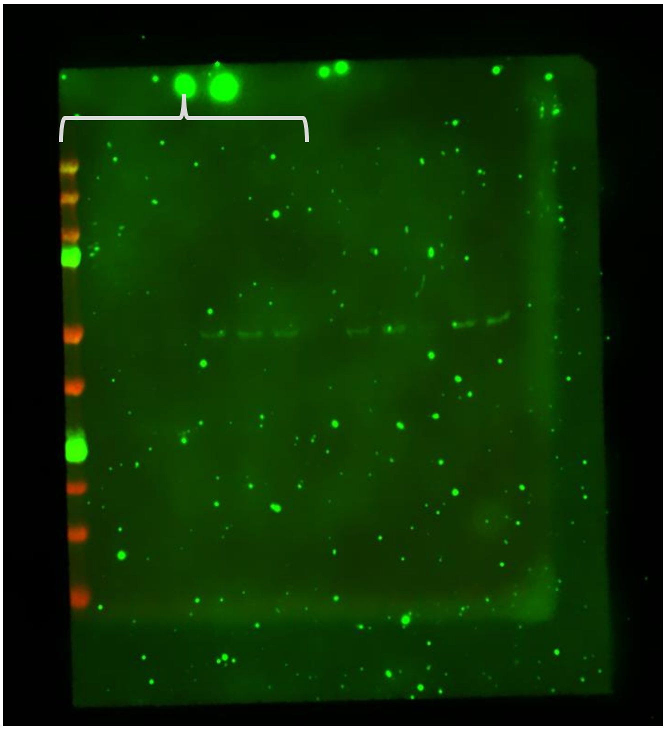

Full unedited gel for
Fig. 5A
IRF9
Ladder and
subsequent 6 lanes
included in Fig 5A

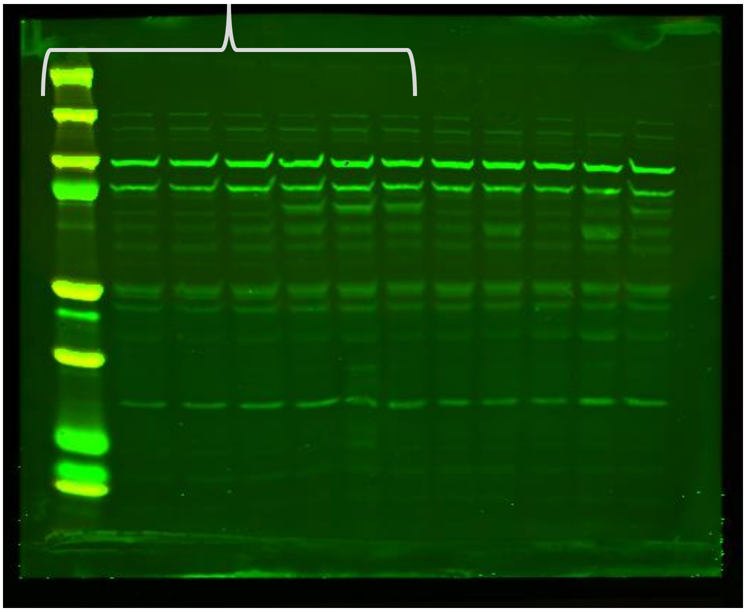

Full unedited gel for
Fig. 5A
OAS2
Ladder and
subsequent 6 lanes
included in Fig 5A

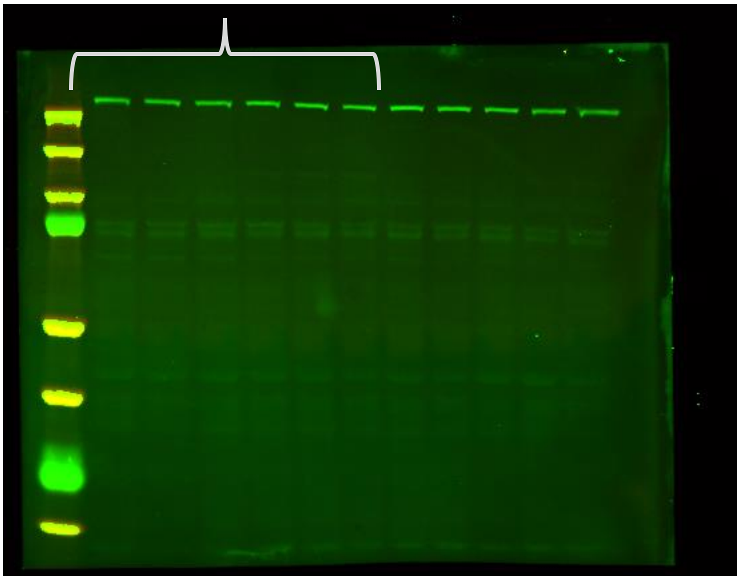

Full unedited gel for
Fig. 5A
OAS3
Ladder and
subsequent 6 lanes
included in Fig 5A

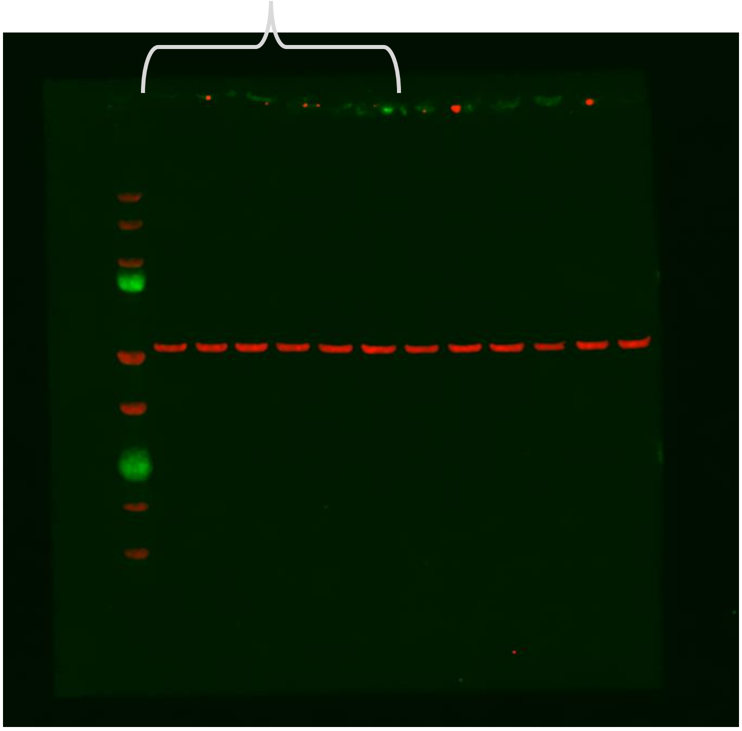

Full unedited gel for
Fig. 5A
α-tubulin
Ladder and
subsequent 6 lanes
included in Fig 5A

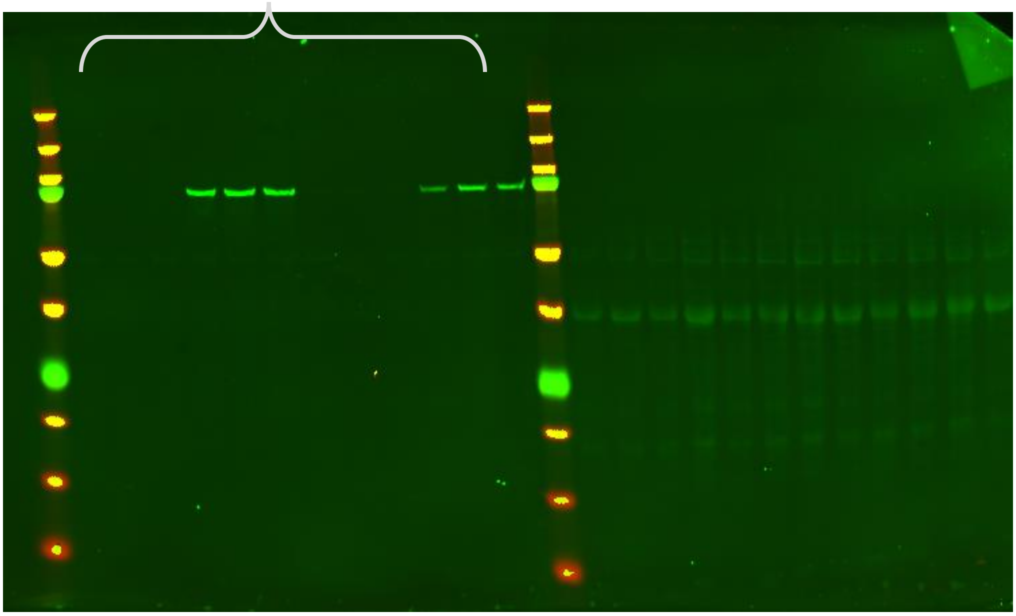

Full unedited gel for Fig. 6B MX1 Ladder and subsequent 11 lanes included in Fig 6B

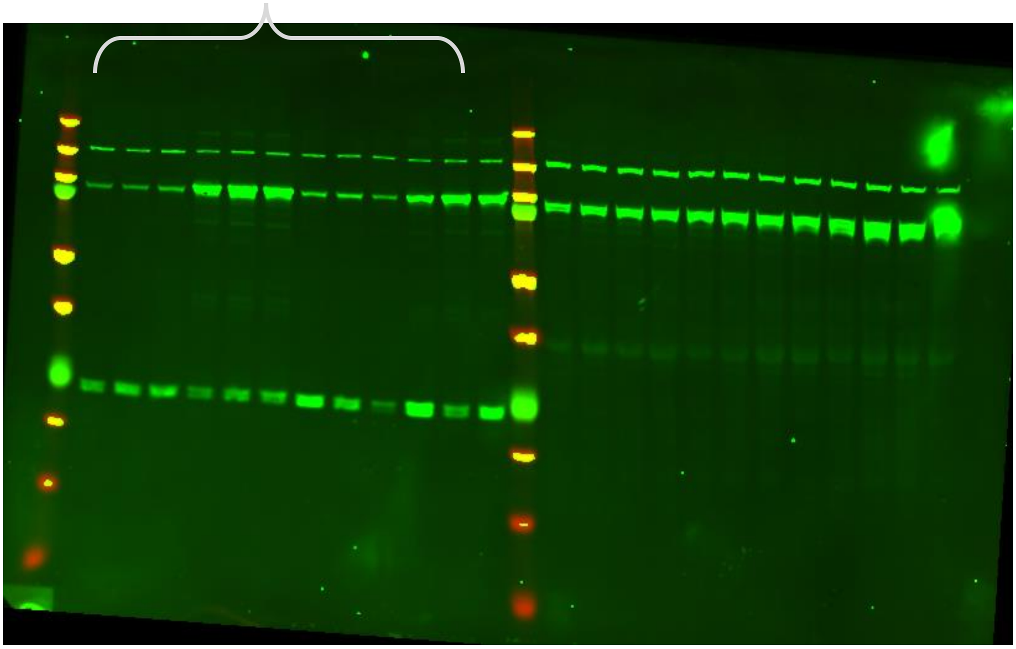

Full unedited gel for Fig. 6B STAT1 Ladder and subsequent 11 lanes included in Fig 6B

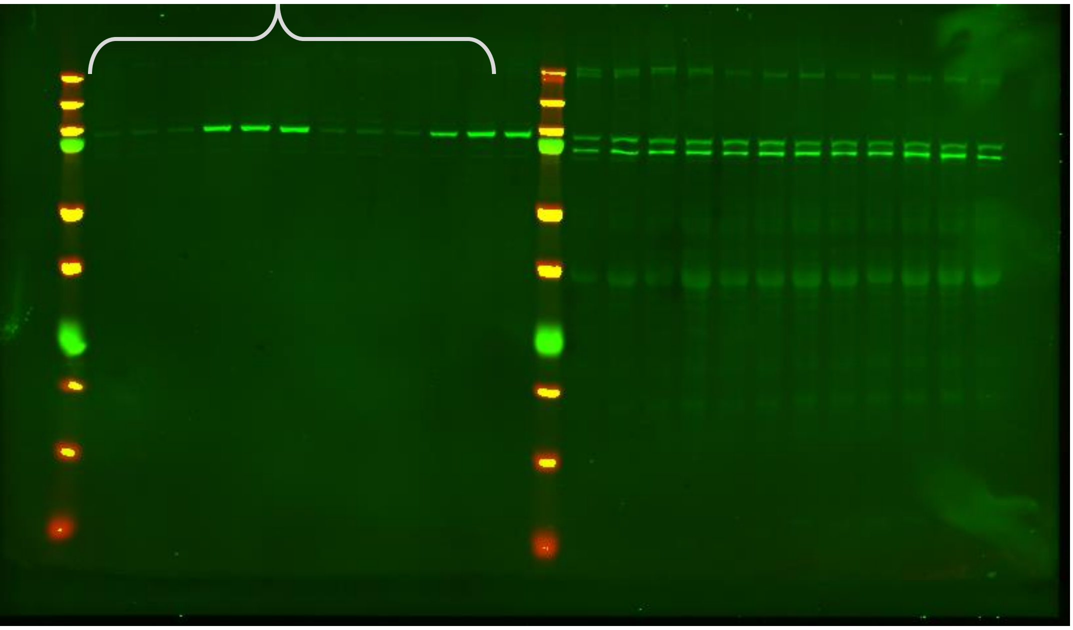

Full unedited gel for Fig. 6B pSTAT1 Ladder and subsequent 11 lanes included in Fig 6B

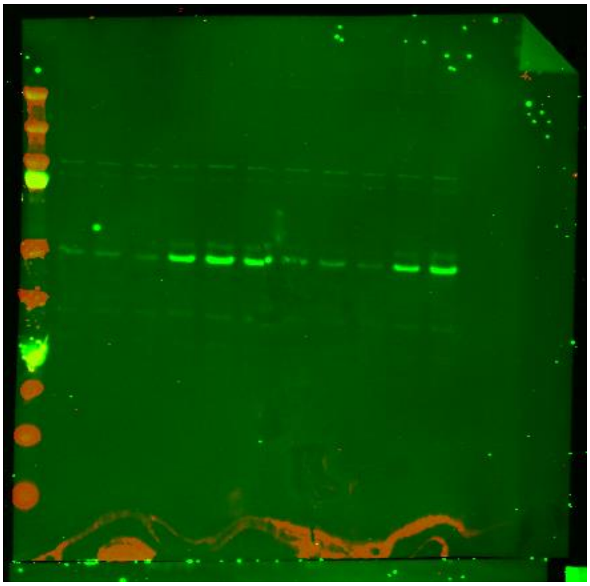

Full unedited gel for Fig. 6B
IRF9
Ladder and subsequent 11 lanes included in Fig 6B

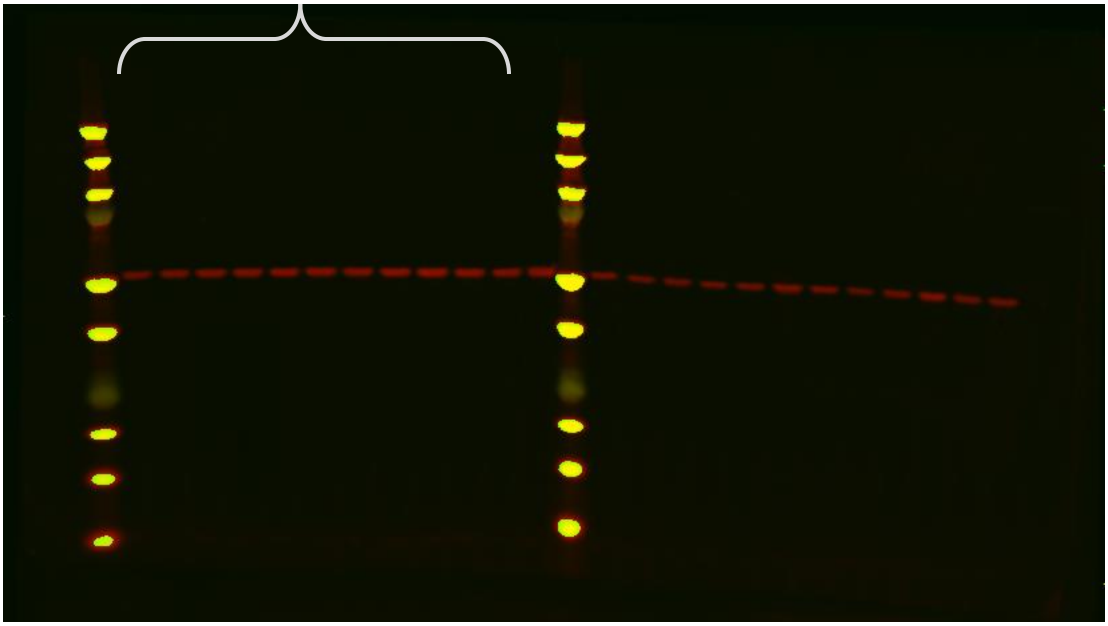

Full unedited gel for Fig. 6B
α-tubulin
Ladder and subsequent 11 lanes included in Fig 6B

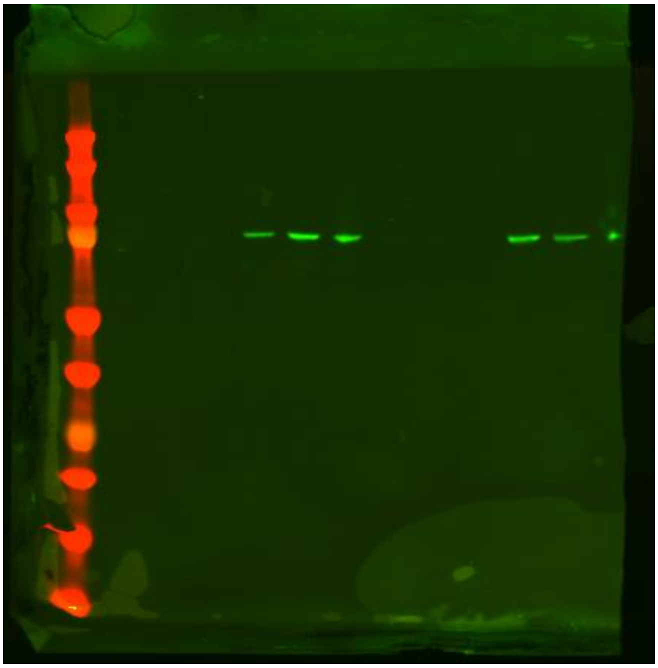

Full unedited gel for Fig. 6C
MX1
Ladder and subsequent 11 lanes included in Fig 6C

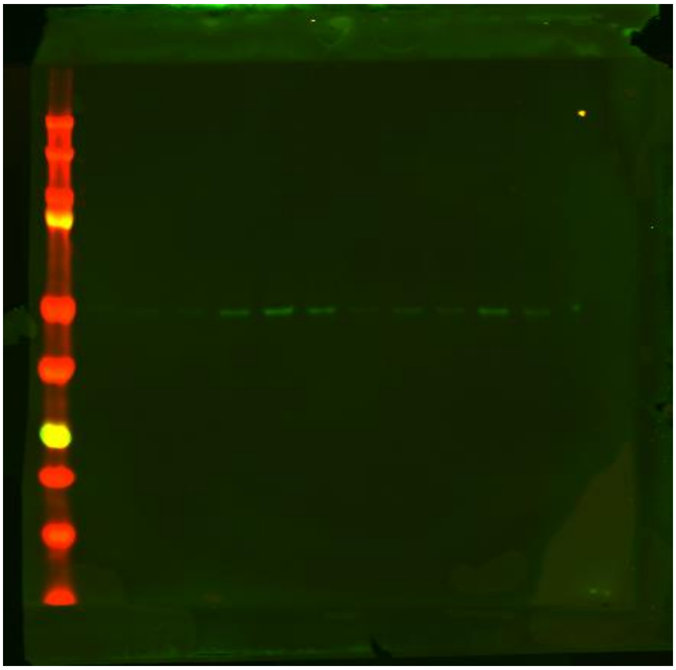

Full unedited gel for
Fig. 6C
IRF9
Ladder and
subsequent 11 lanes
included in Fig 6C

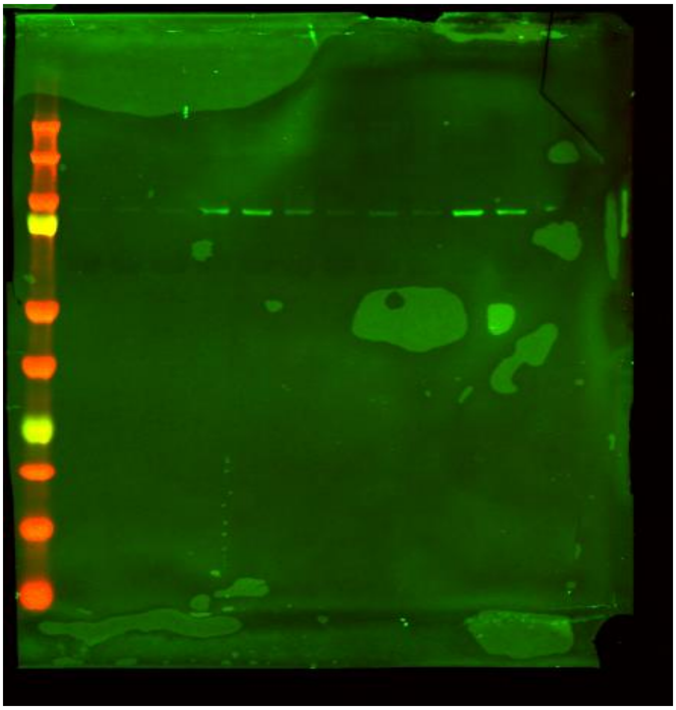

Full unedited gel for
Fig. 6C
pSTAT1
Ladder and
subsequent 11
lanes included in Fig
6C

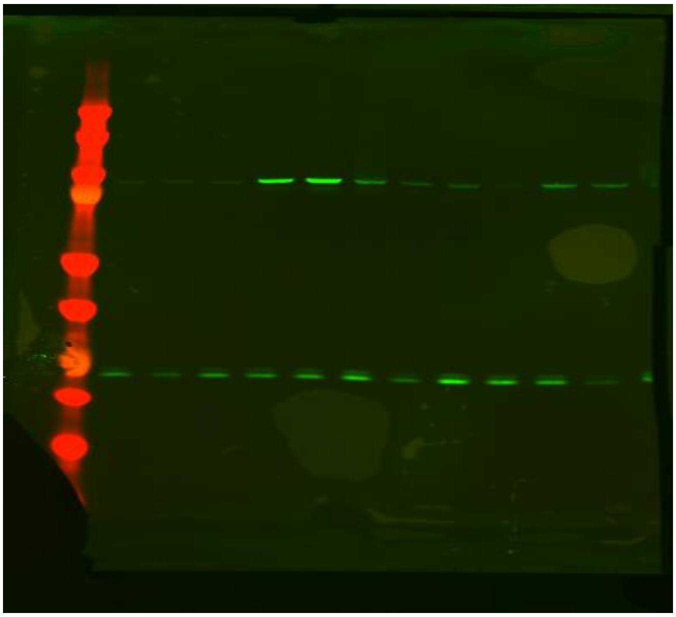

Full unedited gel
for Fig. 6C
STAT1
Ladder and
subsequent 11
lanes included in
Fig 6C

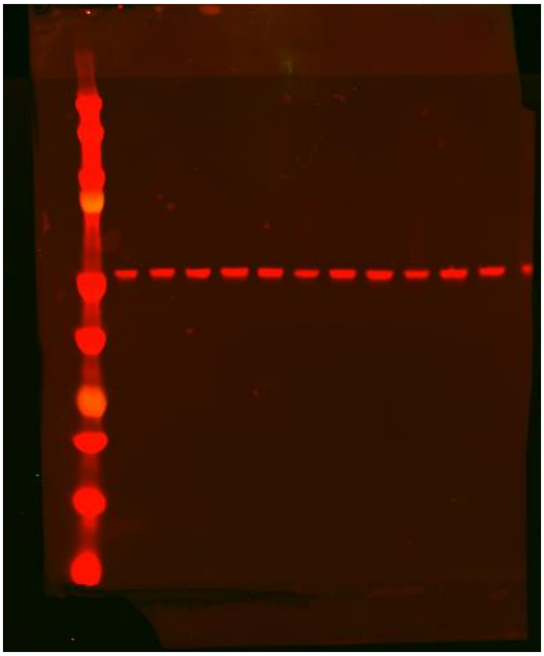

Full unedited gel for Fig. 6C
α-tubulin
Ladder and subsequent 11 lanes included in Fig 6C

Western blot control gels

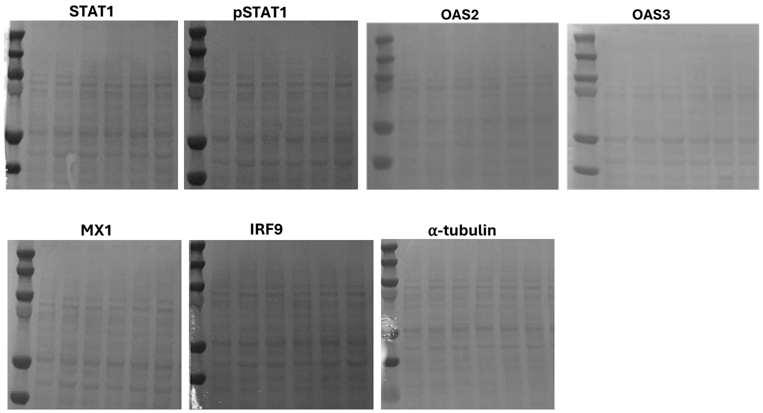

Ancillary data: Total protein loading for western blots shown in Fig.5A of main manuscript. Above figure shows ponceau staining from iPSC-CM cell lysates. Proteins probed from these blots are shown.

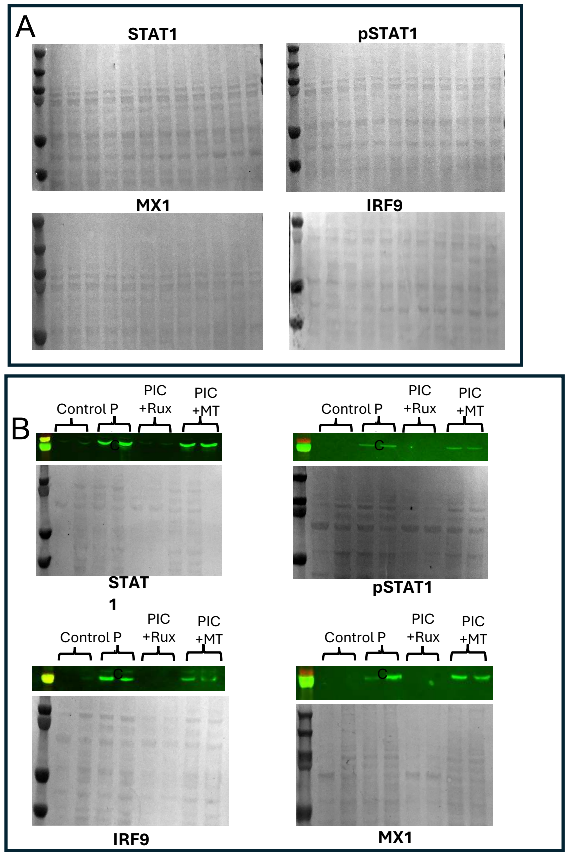

Ancillary data: **(A)**Total protein loading for western blots shown in Fig.6B of main manuscript. Above figure shows ponceau staining from iPSC-CM cell lysates. Proteins probed from these blots are shown. **(B)** Additional blots used to construct bar graphs for fig. 6B in the main manuscript

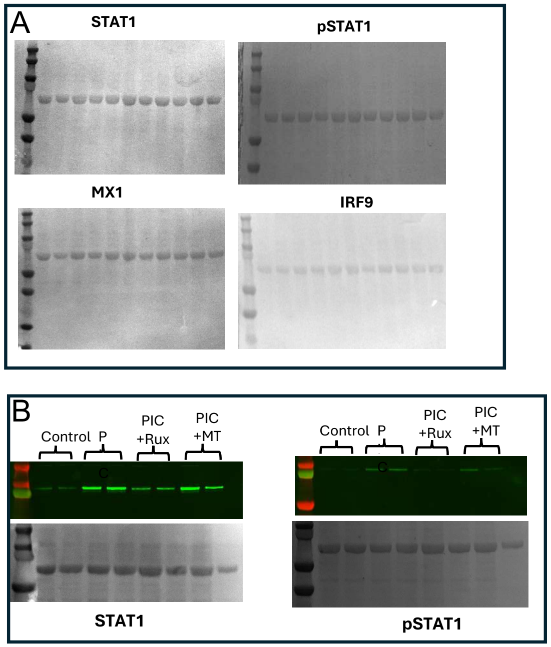

Ancillary data: **(A)**Total protein loading for western blots shown in Fig.6C of main manuscript. Above figure shows ponceau staining from Engineered Heart Tissue cell lysates. Proteins probed from these blots are shown. **(B)** Additional blots used to construct bar graphs for fig. 6C in the main manuscript
